# Depletion of *ZBTB38* potentiates the effects of DNA demethylating agents in cancer cells via *CDKN1C* mRNA up-regulation

**DOI:** 10.1038/s41389-018-0092-0

**Published:** 2018-10-11

**Authors:** Claire Marchal, Maud de Dieuleveult, Claude Saint-Ruf, Nadège Guinot, Laure Ferry, Sara T. Olalla Saad, Mariana Lazarini, Pierre-Antoine Defossez, Benoit Miotto

**Affiliations:** 1 INSERM, U1016, Institut Cochin, Paris, France; 2 CNRS, UMR8104, Paris, France; 3 Université Paris Descartes, Sorbonne Paris Cité, Paris, France; 4 Université Paris Diderot, Sorbonne Paris Cité, Epigenetics and Cell Fate, UMR 7216 CNRS, 75013 Paris, France; 5 Hematology and Blood Transfusion Center-University of Campinas/Hemocentro-Unicamp, Instituto Nacional de Ciência e Tecnologia do Sangue, Campinas, Brazil; 6 Department of Biological Sciences, Federal University of São Paulo, Diadema, Brazil; 7 Present Address: Department of Biological Science, Florida State University, Tallahassee, FL 32306-4295 USA

## Abstract

DNA methyltransferase inhibitor (DNMTi) treatments have been used for patients with myelodysplastic syndromes (MDS) and acute myeloid leukemia (AML), and have shown promising beneficial effects in some other types of cancers. Here, we demonstrate that the transcriptional repressor *ZBTB38* is a critical regulator of the cellular response to DNMTi. Treatments with 5-azacytidine, or its derivatives decitabine and zebularine, lead to down-regulation of ZBTB38 protein expression in cancer cells, in parallel with cellular damage. The depletion of *ZBTB38* by RNA interference enhances the toxicity of DNMTi in cell lines from leukemia and from various solid tumor types. Further we observed that inactivation of *ZBTB38* causes the up-regulation of *CDKN1C* mRNA, a previously described indirect target of DNMTi. We show that *CDKN1C* is a key actor of DNMTi toxicity in cells lacking ZBTB38. Finally, in patients with MDS a high level of *CDKN1C* mRNA expression before treatment correlates with a better clinical response to a drug regimen combining 5-azacytidine and histone deacetylase inhibitors. Collectively, our results suggest that the ZBTB38 protein is a target of DNMTi and that its depletion potentiates the toxicity of DNMT inhibitors in cancer cells, providing new opportunities to enhance the response to DNMT inhibitor therapies in patients with MDS and other cancers.

## Introduction

Vidaza (5-azacytidine), decitabine (5-aza-2-deoxy-cytidine), and zebularine (2(1 H)-pyrimidinone riboside) belong to a class of cytosine analogues that were developed as inhibitors of DNA methylation. The incorporation of these analogues into the DNA (and/or RNA) leads to the formation of covalent bond between the nucleoside analogue and the cysteine thiolate in the catalytic site of the DNA methyltransferases (DNMTs) that establish and maintain DNA methylation patterns during development. This phenomenon eventually leads to the sequestration of the DNMTs, their depletion in the cell, and the passive demethylation of the genomic DNA during DNA replication^1–4^.

5-azacytidine and decitabine have been used to improve survival and health quality of patients with myelodysplastic syndromes (MDS), acute myelogenous leukemia (AML) and chronic myelomonocytic leukemia (CMML)^4–6^. Nonetheless, due to their incorporation into the DNA and the formation of DNA adducts these drugs may have unwanted side effects, limiting their clinical applications^4,7^. There is thus need to develop new therapeutic strategies (i.e., new DNMT inhibitors) and to identify biomarkers that may help predict which patient will most benefit from DNMTi therapy. Several genetic studies have shown that the toxicity and the clinical response of 5-azacytidine derivatives in patients with MDS and AML is influenced by the genetic context^8,9^. Mutations in *TP53*, *TET2*, *IDH1*, *IDH2*, *GADD45A*, and *DNMT3A* correlate with better or poorer drug response in MDS and AML patients^10–17^. At the transcriptional level, expression of *PLBC1*, *BCL2L10* or *micro-RNA-126* influence the response to DNMTi^18–20^. Furthermore, the efficacy of 5-azacytidine can be further enhanced by combination with other compounds including histone deacetylase inhibitors (HDACi)^1,4,7,21^.

The reasons of the toxicity, as well as the mechanism of action of DNMTi, remain not yet fully understood. DNMTi cause passive demethylation of the genomic DNA during DNA replication, coincident with cell proliferation defects and changes in gene expression^2,3,22,23^. Yet, different DNMT inhibitors have variable impact on gene expression, cellular processes and cell death on similar tumor types, questioning the existence of additional effects on protein synthesis, chromatin structure regulation and cell death pathways^3,14,21–23^. For instance, depletion of transcription factor p53 in embryonic fibroblasts from mice strongly enhances the cytotoxicity of 5-azacytidine treatments by potentiating a deadly interferon response^24^. A similar phenomenon has been documented in human ovarian cancer cells exposed to decitabine^15,25^.

Herein, we hypothesized that DNMTi might have an effect on the transcription factors that bind methylated DNA, so we evaluated the impact of 5-azacytidine on the function and expression of the zinc finger and BTB domain containing protein ZBTB38, that binds to methyl-CpGs^26–28^. *ZBTB38* is involved in various cellular functions, including the regulation of DNA replication, the control of gene expression and the regulation of cell proliferation and differentiation^26,29–32^. We observed that 5-azacytidine causes the down-regulation of ZBTB38 protein expression. In addition, we demonstrated that the depletion of *ZBTB38*, or its regulator deubiquitinase USP9X, enhances the cytotoxicity of 5-azacytidine derivatives in different cancer and leukemia cells, which was concomitant with the enhanced expression of *CDKN1C* mRNA. Finally, we observed a correlation between *CDKN1C* mRNA expression in MDS patients and the clinical response to a combination of 5-azacytidine and HDACi. Altogether our work suggests that inhibition (or inactivation) of *ZBTB38* or *USP9X* expression may be a new strategy to enhance the clinical efficacy of DNMTi in hematological and non-hematological cancers.

## Results

### 5-azacytidine causes a decrease of ZBTB38 protein abundance

**Fig. 1:**
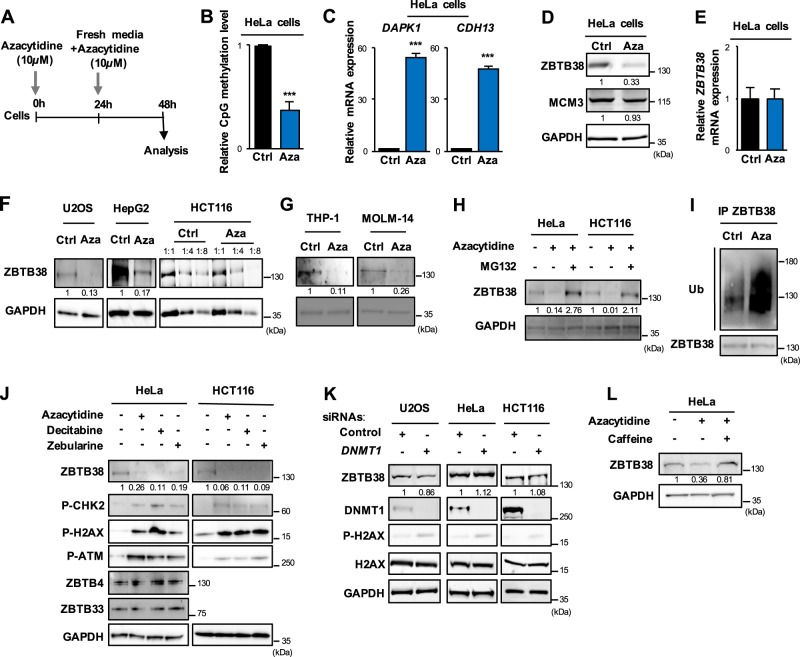
DNA hypomethylating agents cause ZBTB38 down-regulation at protein level in solid and hematologic cancer cells. **a** Schematic representation of the experimental protocol. Cancer cells were treated twice with 10 µM 5-Azacytidine (Aza) or DMSO and harvested 24 h later for analysis. **b** Dot blot analysis of CpG methylation on preparation of genomic DNA from cells treated or not with 5-azacytidine (*n* = 2). **c** Gene expression analysis of hyper-methylated silenced genes *CDH13* and *DAPK1* by real-time PCR in HeLa cells treated with 5-azacytidine (Aza) compared to control (Ctrl) cells (*n* = 3). Expression level is expressed as the fold change between treated and control cells. **d** Western blot analysis of ZBTB38, MCM3, and GAPDH protein expression in HeLa cells treated with 5-azacytidine (Aza) compared to control (Ctrl) cells. **e** Gene expression analysis of *ZBTB38* by real-time PCR in HeLa cells treated with 5-azacytidine (Aza) compared to control (Ctrl) cells (*n* = 3). **f** Western blot analysis of ZBTB38 and GAPDH protein expression in U2OS, HepG2, and HCT116 cancer cells treated with 5-azacytidine (Aza) compared to control (Ctrl) cells. **g** Western blot analysis of ZBTB38 and GAPDH protein expression in THP-1 and MOLM-14 AML cells treated with 5-azacytidine (Aza) compared to control (Ctrl) cells. **h** Western blot analysis of ZBTB38 and GAPDH protein expression in HeLa and HCT116 cancer cells treated with 5-azacytidine (Aza), 5-azacytidine plus proteasome-inhibitor MG132 (4 h) compared to control cells. **i** Western blot analysis of ZBTB38-ubiquitination in HCT116 cells treated with 5-azacytidine (Aza) compared to control (Ctrl). Co-immuno-precipitates of ZBTB38 were run on a SDS-page and ubiquitin-moieties detected using a specific antibody. **j** Western blot analysis of ZBTB38, ZBTB4, ZBTB33, GAPDH, phospho-CHK2 (P-CHK2), phospho-H2AX (P-H2AX) and phospho-ATM (P-ATM) in HeLa and HCT116 cells treated with 10 µM of either 5-azacytidine, decitabine or zebularine and in control mock-treated cells. **k** Western blot analysis of ZBTB38, DNMT1, phospho-H2AX (P-H2AX), H2AX, and GAPDH protein levels in different human cell lines (HeLa, U2OS, HCT116) treated with a specific siRNA against *DNMT1* or a control siRNA**. l** Western blot analysis of ZBTB38 and GAPDH in HeLa cells treated with 10 µM of 5-azacytidine alone or co-treated with caffeine (1 mM) for 24 h

Transcription factor ZBTB38 binds with high affinity to DNA sequences containing methylated CpG sites in vitro, and is recruited at hyper-methylated peri-centromeric sequences in murine cells^27–30,33^. We thus decided to further explore the relationship between ZBTB38 and DNA methylation and tested whether alteration of DNA methylation pattern would interfere with the function of ZBTB38. We exposed human HeLa cells to 5-azacytidine for 48 h (Fig. 1a), which led to global loss of CpG methylation (Fig. 1b). We further confirmed the loss of methylation by showing that hyper-methylated genes (*CDH13* and *DAPK1*) were highly expressed in 5-azacytidine treated samples compared to control samples (Fig. 1c). Western blot analysis showed that ZBTB38 expression was lower in 5-azacytidine-treated whole cell protein extracts compared to untreated cells (Fig. 1d). Expression of the DNA replication factor MCM3 was used as a negative control and excluded a general impact of 5-azacytidine on the activity of the proteasome or on mechanisms of protein synthesis (Fig. 1d). Differing from protein expression, *ZBTB38* mRNA was expressed at similar levels in 5-azacytidine-treated cells compared to control cells (Fig. 1e). In three additional human cancer types (U2OS, HepG2, and HCT116) and two leukemia cell types (THP-1 and MOLM-14) we also observed that exposure to 5-azacytidine causes the down-regulation of ZBTB38 protein abundance without altering the mRNA level (Fig. 1f, g and Supplementary Fig. S1A, B). The amplitude of ZBTB38 protein down-regulation is variable among the cell types studied (Fig. 1d, f, g). We thus monitored the level of expression of ZBTB38 protein in a set of isogenic cells: HCT116 *DNMT1* and *DNMT3B* knockout (HCT116 DKO), HCT116 *TP53*^−/−^ and parental HCT116 cells treated with 5-azacytidine (Supplementary Fig. S1C, D). In all cell types, 5-azacytidine treatment induced a dose-dependent down-regulation of ZBTB38 protein, but the down-regulation in HCT116 DKO cells was rather modest while it was much stronger in HCT116 *TP53*^−/−^ cells compared to isogenic HCT116 cells (Supplementary Fig. S1C, D). We then investigated the abundance of ZBTB38 in MOLM14 cells (i.e., AML cells p53 positive) transfected with siRNA against p53 or control siRNA and further exposed to azacytidine. The abundance of ZBTB38 was lower in cells transfected with siRNAs against p53 (Supplementary Fig. S1E). In other words, 5-azacytidine causes the down regulation of ZBTB38 protein expression in a variety of cancer and leukemia cells and this regulation is affected by the cellular background of the cell, notably the status of *DNMT1/3B* and *TP53*.

ZBTB38 is regulated by poly-ubiquitination and proteasome degradation^30–32^. We thus investigated the implication of this pathway in 5-azacytidine induced ZBTB38 protein down-regulation. For this purpose, HeLa and HCT116 cells were concomitantly treated with a proteasome inhibitor (MG132) and 5-azacytidine. We observed that the addition of MG132 induced a higher ZBTB38 expression in comparison to 5-azacytidine alone (Fig. 1h). Further, we immunoprecipitated ZBTB38 from HCT116 cells treated with 5-azacytidine (in presence of MG132). We confirmed that ZBTB38 is poly-ubiquitinated in control cells and observed that the amount of poly-ubiquitination is dramatically increased in 5-azacytidine treated cells (Fig. 1i). In conclusion, 5-azacytidine causes *ZBTB38* down-regulation in different cancer cell types, mainly at the protein level and most likely in a proteasome-dependent manner. We therefore investigated the regulation of E3 ubiquitin ligase RBBP6 and deubiquitinase USP9X that controls ZBTB38 poly-ubiquitination in human cells^31,32^. We observed that RBBP6 protein and mRNA levels were up-regulated upon 5-azacytidine treatment and that this up-regulation occurs at the protein and mRNA levels (Supplementary Fig. S1F, G). No detectable changes in USP9X protein levels were observed at the time point tested (Supplementary Fig. S1H). We tested the hypothesis that *RBBP6* up-regulation may be a consequence of the demethylation of RBBP6 promoter. Using immunoprecipitation of methylated DNA, we could not detect significant levels of DNA methylation at the RBBP6 promoter (Supplementary Fig. S1I), arguing against a direct effect of 5-azacytidine on the methylation of the *RBBP6* promoter. These data indicate that the down-regulation of ZBTB38 protein levels upon 5-azacytidine treatment is mediated by an ubiquitination pathway and most likely occurs through the up-regulation of E3 ubiquitin ligase RBBP6.

### Down-regulation of ZBTB38 protein level coincides with 5-azacytidine cytotoxicity

Azacytidine mechanism of action is associated with its DNA demethylating activity but also with indirect cytotoxic effects^2,15,22–25,34^. We thus monitored ZBTB38 protein expression in three conditions: in cells treated with different DNMT inhibitors, in cells genetically inactivated for the enzymes involved in the establishment and maintenance of DNA methylation and in cells transfected with DNMT1 RNAi molecules. In parallel, we monitored DNA methylation levels and the presence of DNA damage in the same samples.

Azacytidine, decitabine, and zebularine treated cells presented decreased ZBTB38 protein expression compared to control samples (Fig. 1j), while *ZBTB38* mRNA levels were quite similar between the different samples (Supplementary Fig. S1J). Again, the effect of DNMTi was specific as the expression of ZBTB4 and ZBTB33/KAISO, two paralogs of ZBTB38, was not altered (Fig. 1j). We observed a significant increase in phospho-H2AX, a marker of cellular damage, in DNMTi treated cells compared to vehicle treated cells (Fig. 1j). Cellular damage was confirmed by the increase in phospho-CHK2 and phospho-ATM signals in the same DNMTi treated samples (Fig. 1j). Importantly, cells genetically inactivated for DNMT1 and DNMT3B (HCT116 Double Knock-Out), which lose ~75% of their DNA methylation, did not present changes in ZBTB38 protein abundance compared to isogenic parental cells (Supplementary Fig. S1K, L). This observation indicates that ZBTB38 protein expression was not directly correlated with the steady-state amount of DNA methylation in cells, nor dependent on a physical interaction with DNMT1 or DNMT3B.

DNMT1 is the main enzyme that restores CpG methylation patterns following genome replication, so we investigated the consequence of its depletion on ZBTB38 protein abundance. *DNMT1* silencing was confirmed by western blot after 48 h of transfection in HeLa, U2OS, and HCT116 cells with specific siRNA directed against *DNMT1* (Fig. 1k) and we confirmed that heavily methylated and silenced tumor suppressor genes were reactivated (Supplementary Fig. S2). ZBTB38 protein expression level was similar between *DNMT1*-depleted and control cells (Fig. 1k). In addition, we observed that H2AX phosphorylation was only barely increased in cells transfected with siRNAs against *DNMT1* compared to control cells (Fig. 1k). From this set of experiments, we concluded that the lack of *DNMT1* (or its strong reduction by RNA interference), accompanied by a loss of DNA methylation, does not alter ZBTB38 protein expression. These results indicate that ZBTB38 protein down-regulation by DNMT inhibitors is most likely a consequence of the activation of a stress response pathway or cell death promotion. We thus investigated the level of expression of ZBTB38 in HeLa cells combining DNMTi treatment with caffeine, an inhibitor of ATM-ATR damage-induced kinases. Consistent with our interpretation, we observed that the level of ZBTB38 in cells co-treated with caffeine is significantly higher than in cells treated with azacytidine alone (Fig. 1l).

### Depletion of *ZBTB38* does not increase DNA demethylation by DNMT inhibitors

**Fig. 2:**
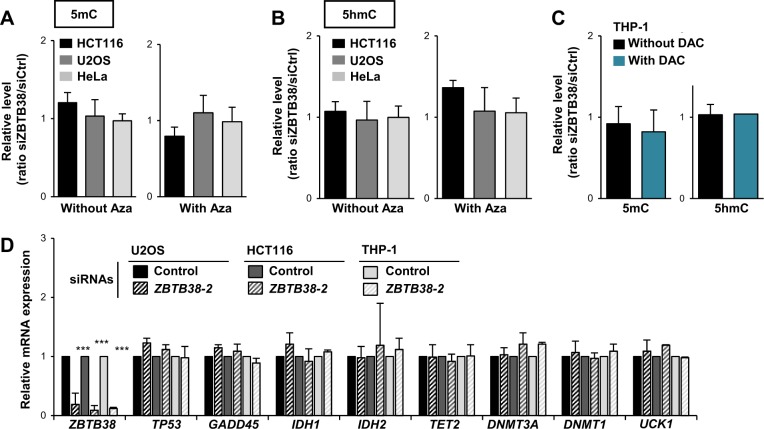
Inactivation of *ZBTB38* marginally impacts DNA demethylation and 5-azacytidine induced DNA demethylation. **a** Dot blot analysis of CpG methylation levels in HCT116 (black bar), U2OS (grey bar), and HeLa (light grey bar) cells treated with siRNA against *ZBTB38* or control siRNA and further exposed to 5-azacytidine (*n* = 3). The data represent relative levels of the condition siRNA *ZBTB38* versus siRNA control. **b** Dot blot analysis of CpG hydroxymethylation levels in HCT116 (black bar), U2OS (grey bar), and HeLa (light grey bar) cells treated with siRNA against *ZBTB38* or control siRNA and further exposed to 5-azacytidine (*n* = 3). The data represent relative levels of the condition siRNA *ZBTB38* versus siRNA control. **c** Dot blot analysis of CpG methylation and hydroxymethylation levels in THP-1 cells treated with siRNA against *ZBTB38* or control siRNA and further exposed to decitabine (*n* = 2). The data represent relative levels of the condition siRNA *ZBTB38* versus siRNA control. **d** RT-PCR analysis of genes modulating 5-azacytidine toxicity in HCT116, U2OS, and THP-1 cells treated with siRNA against *ZBTB38* (dashed bars) and control siRNA (solid bars) (*n* = 3)

We speculated that DNMTi might achieve a higher DNA demethylation in the absence of ZBTB38 protein. To test this hypothesis, we characterized the extent of DNA demethylation induced by DNMTi in *ZBTB38*-silenced cells. The amount of 5-methyl-cytosine (5-mC) and 5-hydroxymethylcytosine (5-hmC), an intermediate in DNA demethylation, was determined on genomic DNA preparations by dot blot. We observed that levels of 5-mC and 5-hmC were similar in cells transfected with control and *ZBTB38* siRNAs as well as in cells combining RNA interference and 5-azacytidine (or decitabine) treatment (Fig. 2a–c). Therefore, depletion of *ZBTB38* by RNA interference does not potentiate the demethylating activity of DNMTi in the tested conditions at the global scale.

We further investigated the expression of enzymes involved in the regulation of DNA methylation, whose mutation and/or expression level were reported to influence 5-azacytidine derivatives sensitivity in AML and MDS patients^10–12,16,17,35^. We evaluated the expression of *DNMT1*, *DNMT3A*, *TET2*, *GADD45A*, *UCK1*, *IDH1* and *IDH2* in HCT116, U2OS and THP-1 cells transfected with control and *ZBTB38* siRNAs (Fig. 2d). All of these genes were expressed at very similar levels in *ZBTB38* depleted cells and control cells (Fig. 2d). In conclusion, depletion of *ZBTB38* does not potentiate the DNA demethylating activity of 5-azacytidine and decitabine, nor does it affect the mRNA expression of the tested set of genes involved in the establishment and/or maintenance of DNA methylation.

### Depletion of *ZBTB38* using specific anti-sense RNA molecules enhances 5-azacytidine cytotoxicity in cancer cells

**Fig. 3:**
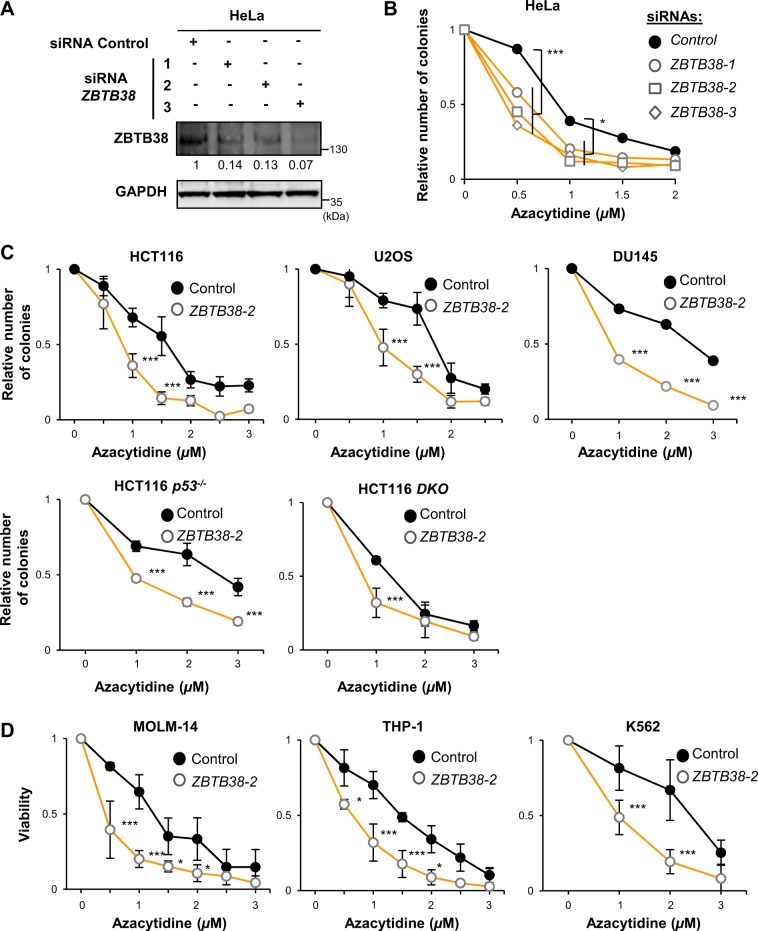
Inactivation of *ZBTB38* by RNA interference enhances the cytotoxicity of 5-azacytidine in a variety of cancer cells. **a** Western blot analysis of ZBTB38 and GAPDH expression in cells treated with three different anti-sense RNA directed against *ZBTB38* and a control anti-sense RNA. Relative expression level of ZBTB38 is indicated under the blots with the control condition set up as 1. **b** Depletion of *ZBTB38* enhances the cytotoxicity of 5-azacytidine in colony formation assays of HeLa cells (*n* = 3). Colony were counted 15 days post-azacytidine exposure and normalized to the condition without 5-azacytidine, artificially set up as 1. ***P < 0.001; *P < 0.05. **c** Depletion of *ZBTB38* enhances the cytotoxicity of 5-azacytidine in colony forming assays of HCT116, U2OS, DU145, HCT116 *p53*^*-*^^−/−^ and HCT116 *DKO* cells (*n* = 4). ***P < 0.005. **d** Depletion of *ZBTB38* enhances the cytotoxicity of 5-azacytidine in leukemia K562, THP-1, and MOLM-14 cells (*n* = 3). Cell viability was analyzed 2 days post-azacytidine exposure by scoring trypan blue-positive cells and reported as the number of viable cells normalized to the condition without 5-azacytidine, artificially set up as 1. ***P < 0.001; *P < 0.05

We tested whether *ZBTB38* inactivation would modulate the cytotoxicity of 5-azacytidine in cancer cells. Cells were transfected with siRNAs molecules directed against *ZBTB38* and 48 h later 5-azacytidine (concentration ranging from 1 to 10 µM) was added to the culture media for 48 h. Fresh media (without drug) was then replaced every 3 days for two weeks and cell colony counting was performed at that stage. We observed that depletion of *ZBTB38* with specific siRNAs causes a significant inhibition in the formation of HeLa colonies at different concentrations of 5-azacitidine (Fig. 3a, b). This effect was confirmed in other cancer cell types, notably HCT116, HCT116 *TP53*^−/−^, HCT116 DKO, U2OS, and DU145 (Fig. 3c). We also monitored the proliferation of leukemia cells: K562, THP-1, and MOLM-14. Cells were transfected with siRNA molecules and 48 h later exposed to 5-azacytidine for 48 h. Live cell counting was performed at that stage (Fig. 3d). Again the depletion of *ZBTB38* enhanced the deleterious effects of 5-azacytidine and the amplitude of the cytotoxicity caused by *ZBTB38* depletion was variable among the different cell types.

We then extended the analysis to decitabine and zebularine on a panel of cell types. On its own, decitabine inhibits cell growth in a concentration dependent manner (Supplementary Fig. S3A). Depletion of *ZBTB38* further enhanced the cytotoxicity of decitabine in all tested cell types (Supplementary Fig. S3A). Zebularine barely impact cell growth even at the highest concentration we tested (Supplementary Fig. S3B). However, *ZBTB38* silencing significantly inhibits colony formation in the presence of zebularine, in the two cell types tested (Supplementary Fig. S3B). Altogether, our results demonstrate that depletion of *ZBTB38* enhances the cytotoxicity of several DNMT inhibitors in various cancer cells.

In order to further demonstrate that the depletion of *ZBTB38* enhances the cytotoxicity to DNMTi, we compared the colony formation capacity of HeLa and U2OS cells when ZBTB38 depletion and decitabine exposure are simultaneous and when ZBTB38 depletion is performed after decitabine exposure. We observed that the number of colonies was similar between control and ZBTB38-depleted cells when ZBTB38 depletion is performed after decitabine exposure (Supplementary Fig. S3C–E). This result indicates there is no synergistic effect when cells are exposed to DNMTi prior to ZBTB38 depletion, supporting our hypothesis that DNMTi cytotoxic effects should be at least partially mediated by *ZBTB38*.

### Transient depletion of ZBTB38 or USP9X combined with decitabine exposure causes a long-term arrest of leukemia cell proliferation

**Fig. 4:**
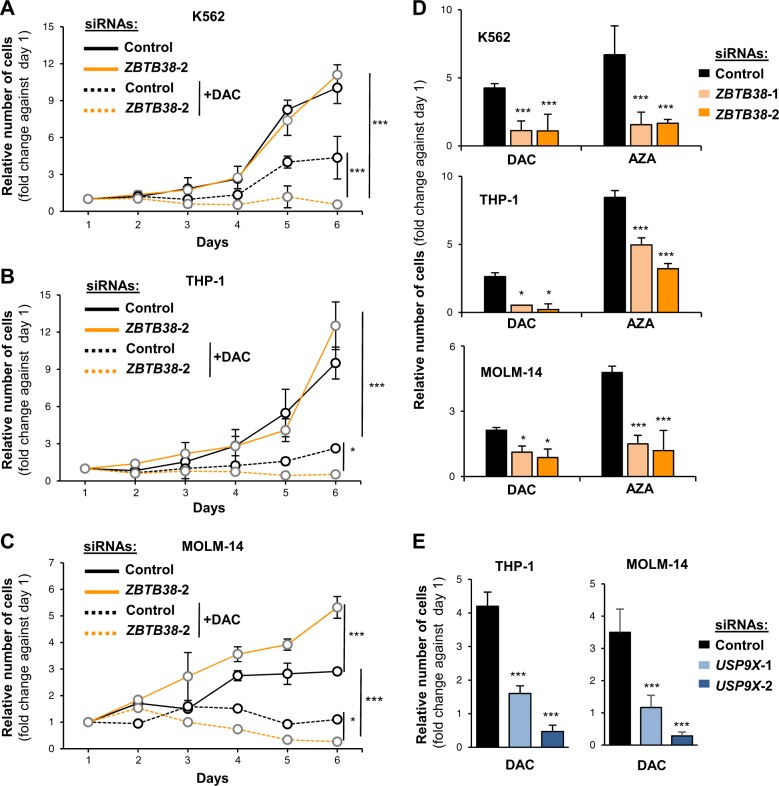
Inactivation of *ZBTB38* by RNA interference causes a strong arrest in cell proliferation upon DNMTi exposure, persistant after removal of DNMTi. **a** Graph depicting the growth curve of K562 cells treated with siRNAs (control and ZBTB38) and with decitabine 1 µM (DAC) or not (*n* = 4). ***P < 0.001. **b**, **c** Same experiment in THP-1 (**b**) and MOLM-14 (**c**). **d** Graph depicting the proliferative potential of THP-1, MOLM-14, and K562 cells transfected with siRNAs (control and ZBTB38) and further exposed to decitabine (DAC) or azacytine (AZA) (*n* = 4). ***P < 0.001. *P < 0.05. **e** Graph depicting the proliferative potential of THP-1 (left panel) and MOLM-14 (right panel) cells transfected with siRNAs (control and USP9X) and further exposed to decitabine 1 µM (DAC) (*n* = 4). ***P < 0.001

We investigated the kinetics of cell proliferation when ZBTB38 depletion is combined with decitabine or azacytidine exposure in leukemia cells. We treated K562, THP-1, and MOLM-14 cells with decitabine (1 µM, a dose achieved in human plasma^3^ for 24 h. After this period, the cells were washed with fresh media (without decitabine) and proliferation was monitored for 6 days. Under normal condition of cell culture, inactivation of ZBTB38 by RNA interference barely impacted the proliferation of K562 and THP-1 cells, while it stimulated the proliferation of MOLM-14 cells (Fig. 4a–c). Treatment with decitabine causes a strong inhibition of cell proliferation but cells eventually tend to regain cell proliferative abilities on day 4 or 5 post-decitabine treatment depending on the cell type (Fig. 4a–c). On the contrary, ZBTB38-depleted cells were not capable to regain proliferative abilities until the end of the experiment (Fig. 4a–c). We thus concluded that the transient depletion of ZBTB38 at the time of decitabine exposure negatively impact leukemia cell proliferation even after the return to the normal culture conditions.

We next tested the consequences of *ZBTB38* depletion and azacytidine treatment on cell proliferation. We observed a stronger inhibition of cell proliferation when azacytidine treatment is combined with *ZBTB38* silencing compared to azacytidine alone (Fig. 4d). The defect in cell proliferation is even more pronounced than upon decitabine treatment and it is recapitulated with two different siRNAs against *ZBTB38* (Fig. 4d). These observations indicate that the transient silencing of *ZBTB38* at the time of DNMTi treatment negatively affects the proliferation of leukemia cells.

We further supported this conclusion using two additional approaches. We tested the consequences of *USP9X* depletion and decitabine exposure on cell proliferation. We transfected THP-1 and MOLM-14 cells with two different siRNAs against *USP9X* and treated the cells with decitabine (1 µM) for 24 h. After this period, the cells were washed with fresh media (without decitabine) and proliferation was monitored for 7 days. Without DNMTi, inactivation of *USP9X* by RNA interference barely impacted the proliferation of MOLM-14 cells, while it stimulated the proliferation of THP-1 cells (Supplementary Fig. S4A-B). USP9X depleted cells were barely able to proliferate in presence of DAC compared to control cells that grow very slowly (Fig. 4e and Supplementary Figure S4A-B). The expression of ZBTB38 was very low in both cell lines silenced for USP9X compared to control, further supporting that low ZBTB38 protein level enhances DNMTi toxicity (Supplementary Fig. 4A-B). In a second experiment we utilized a different protocol of DAC exposure. We used lower levels of DAC (0.1 µM) that was renewed every day for 5 days. In this experimental set up, we also observed that cells depleted of ZBTB38 or USP9X proliferate slowly than control siRNA transfected cells (Supplementary Fig. S4C). This suggests that depletion of ZBTB38 or its deubiquitinase USP9X enhances DAC toxicity in THP1 and MOLM-14 cells independently of DAC exposure protocol.

### Depletion of *ZBTB38* causes a significant increase in cell death upon DNMTi exposure in solid and hematologic cancer cells

We conducted an analysis of the cell cycle, cell death, and autophagy by flow cytometry in HeLa and THP-1 cells transfected with or without ZBTB38 siRNAs and further exposed or not to decitabine. We could not find evidence of significant changes in autophagy and cell cycle progression in cells combining *ZBTB38* depletion and DNMTi exposure compared to DNMTi alone (Supplementary Fig. S5A–E). We however observed a modest increased in cell death in cells combining *ZBTB38* depletion and decitabine exposure. This was mostly noticeable by an increase in necrotic cells (positive for propidium iodide and negative for annexin V) and by the presence of cell debris (Supplementary Fig. S5F–H).

### Depletion of *ZBTB38* increases the expression of *CDKN1C* at mRNA level

**Fig. 5:**
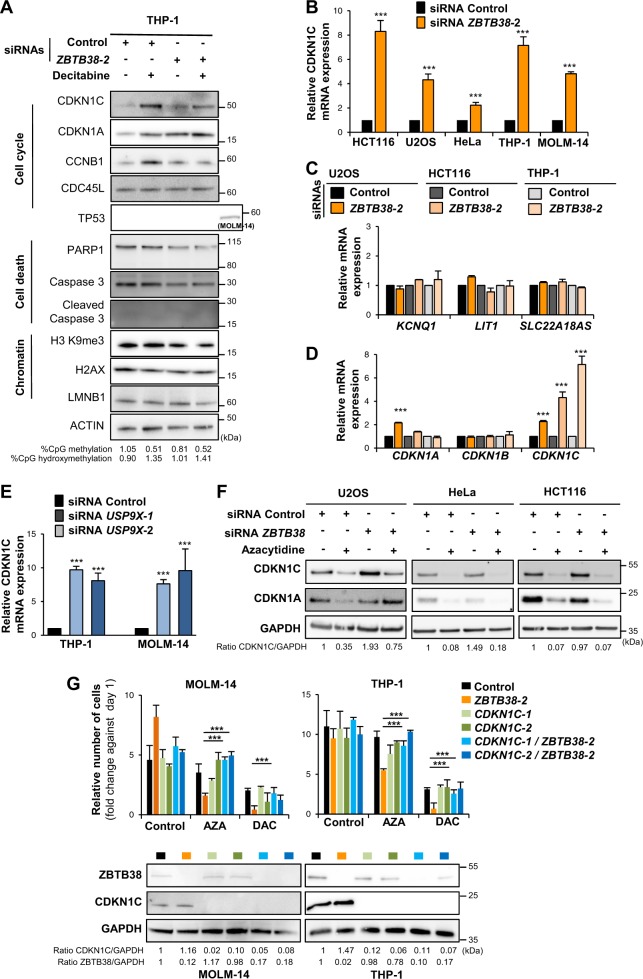
Depletion of *ZBTB38* induces *CDKN1C* up-regulation at the mRNA level in solid and hematologic cancers. **a** Western blot analysis of cell death, cell cycle, and DNA replication factors expression in THP-1 cells treated with siRNAs (control and *ZBTB38*) and with decitabine (1 µM for 24 h) or not. The experiment was performed on day 1. Levels of 5 mC and 5 hmC evaluated by dot blot are indicated. TP53 expression in MOLM-14 cells is also presented as control. **b** Analysis by RT-qPCR of *CDKN1C* expression in U2OS, HCT116, HeLa, THP-1, and MOLM-14 cells treated with an siRNA against *ZBTB38* and control siRNA (*n* = 3). **c** RT-qPCR analysis of the expression of genes located in the vicinity of *CDKN1C* at the ICR1 locus in HeLa, HCT116, and THP-1 cells (*n* = 3). **d** Analysis by RT-qPCR of *CDKN1A*, *CDKN1B*, and *CDKN1C* expression levels in HeLa, HCT116, and THP-1 cells treated with an siRNA against *ZBTB38* and control siRNA (*n* = 3). **e** Analysis of *CDKN1C* expression by RT-qPCR in THP-1 and MOLM-14 cells transfected with siRNA against *USP9X* or control siRNA (*n* = 3). **f** Western blot analysis of CDKN1C protein expression in U2OS, HeLa, and HCT116 cells challenged with 5-azacytidine (4 µM for 24 h) and further treated with siRNAs against *ZBTB38*. **g** Depletion of *CDKN1C* rescues the cytotoxicity caused by DNMTi and ZBTB38 silencing in THP-1 and MOLM-14 cells (*n* = 3). Cell viability was analyzed 2 days post-DNMTi exposure (azacytidine: 1 µM for 24 h and decitabine: 2 µM for 24 h) by scoring trypan blue-positive cells and by reporting the ratio between the number of viable cells at Day 2 compared to Day 0. ***P < 0.05. A representative western blot of CDKN1C and ZBTB38 protein expression in each condition is shown for one of the three replicate

We analyzed the expression of several markers of cell death and cell cycle arrest by western blot in cellular extracts prepared from THP-1 cells transfected with or without ZBTB38 siRNAs and further exposed or not to decitabine. We investigated the proteolytic cleavage of PARP-1 and Caspase3. Consistent with FACS analyses we could not observe increased cleavage of PARP-1 and Caspase3 in cells combining ZBTB38 depletion and decitabine compared to all other experimental conditions (Fig. 5a). Among all the other factors investigated, we observed an alteration in CDKN1C (also known as p57 and KIP2), CDKN1A (p21 or WAF1), and CCNB1 (Cyclin B1) protein expression levels (Fig. 5a). CDKN1C and CCNB1 expression levels were higher in *ZBTB38* depleted cells compared to control cells under normal culture conditions (Fig. 5a). Differing from untreated conditions, CDKN1C and CCNB1 protein expression levels were lower in cells combining *ZBTB38* depletion and decitabine exposure compared to decitabine alone. CDKN1A/p21 expression was higher in cells depleted of *ZBTB38*, exposed to decitabine and combining both treatments compared to control (Fig. 5a). These findings indicated that specific cell pathways might be altered when *ZBTB38* depletion is combined with DNMTi exposure.

We further focused our study on CDKN1C and ZBTB38 for three main reasons. First, *CDKN1C* is known for its role in the regulation of cell growth, cellular senescence, and MDS development^36,37^. Second, *CDKN1C* is an indirect transcriptional target of azacytidine and decitabine in multiple cancer cells^1,3,22^. Third, ZBTB38 paralogs, ZBTB4 and ZBTB33, regulate the expression of CDKN1 family genes in cancer cells^38–41^.

We first investigated the expression of *CDKN1C* mRNA in different cell types treated with siRNAs against *ZBTB38*. By RT-qPCR, we observed that depletion of *ZBTB38* induces an up-regulation of *CDKN1C* mRNA expression in all the cell types tested (Fig. 5b). *CDKN1C* is located in a region of chromosome 11 containing many imprinted genes and long non coding RNAs, with complex direct and indirect transcriptional regulation^42^. We studied the expression of CDKN1C neighboring genes in HeLa and THP-1 cells silenced for *ZBTB38*. *ZBTB38* knock-down did not change *KCNQ1*, *SLC22A18AS* and the long non coding RNA *LIT1* (or *KCNQ1OT*) expression levels compared to control cells (Fig. 5c). We also observed that CDKN1A and CDKN1B mRNA expression levels were marginally affected by the depletion of *ZBTB38* (Fig. 5d). We also investigated CDKN1C mRNA expression in THP1 and MOLM-14 cells treated transfected with two different siRNAs against *USP9X*. We observed the up-regulation of CDKN1C mRNA expression in cells depleted of USP9X compared to control (Fig. 5e).

We then investigated the expression of *CDKN1C* in different cancer cells by western blot (Fig. 5f). Depletion of *ZBTB38* causes CDKN1C protein up-regulation in U2OS and HCT116 cells to different extent, and no significant up-regulation in HeLa cells (Fig. 5f). When cells were further challenged with 5-azacytidine we observed a down-regulation of CDKN1C at protein level in U2OS and HCT116 but the amount of CDKN1C remained higher in the siRNA *ZBTB38* condition compare to control cells (Fig. 5f). Depletion of ZBTB38 causes an up-regulation of CDKN1C mRNA and protein in U2OS, HCT116, and THP-1 cells under normal and DNMTi condition. The lack of correlation between *CDKN1C* mRNA and protein expression levels is not unprecedented^43–45^ and suggests that *ZBTB38* depletion might alter both the transcriptional and post-transcriptional regulation of CDKN1C.

We tested whether CDKN1C silencing alter the response to azacytidine or decitabine in MOLM-14 and THP-1 cells. Following transfection of cells with siRNAs against CDKN1C, we exposed the cells to DNMTi and monitored cell survival over 2 days. Depletion of CDKN1C has a minor, if any, impact on the viability of THP-1 and MOLM-14 cells in absence or in presence of azacytidine and decitabine (Fig. 5g). However, cells co-depleted of ZBTB38 + CDKN1C showed a very similar viability as control cells in absence or in presence of DNMTi (Fig. 5g). These experiments suggest that CDKN1C depletion prevents cell defects caused by ZBTB38 depletion and that CDKN1C up-regulation contributes to ZBTB38 toxicity upon DNMTi treatment.

### High levels of CDKN1C mRNA expression are associated with a better response to a combined 5-azacytidine and entinostat treatment in MDS patients

**Fig. 6:**
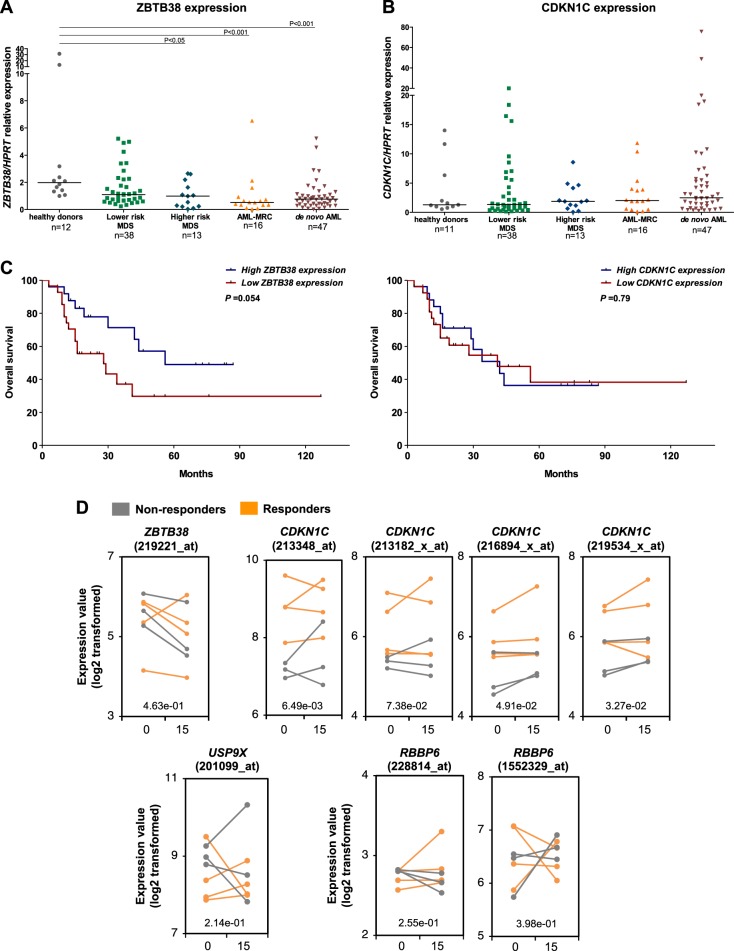
High expression of *CDKN1C* mRNA in the bone marrow of patients with MDS coincides with a better response to 5-azacytidine *plus* entinostat. **a** RT-qPCR analysis of *ZBTB38* and **b** *CDKN1C* mRNA expression in bone marrow samples from patients with MDS, AML with myelodysplasia-related changes (AML-MRC) and de novo AML. MDS patients were grouped into lower and higher-risk categories according to the Revised International Prognostic Scoring System. **c** Kaplan–Meier plots of overall survival in MDS patients classed according to low and high ZBTB38 (left panel) or CDKN1C (right panel) mRNA expression levels. **d** Expression of *ZBTB38*, *RBBP6*, *USP9X*, and *CDKN1C* in samples from patients with MDS before azacytidine and entinostat treatment (D0) and 15 days later (D15). Responders to the therapy are highlighted in orange; non responder are presented in grey (data from ref. ^1^). Significant difference between responder and non-responder at Day 0 is indicated by a *P*-value in the graph

We investigated *ZBTB38* and *CDKN1C* expression in bone marrow samples from MDS and AML patients by quantitative PCR (Fig. 6a, b). Data is showed as median [max–min]. *ZBTB38* expression was decreased in AML with myelodysplasia-related changes (AML-MRC) (0.52 [6.54-0.04]) and in de novo AML (0.77 [5.23–0.01]), compared to healthy donors (1.98 [31.47–1.00]), both *P* < 0.001 (Fig. 6a). When MDS patients were stratified according to Revised International Prognostic Scoring System (IPSS-R) in lower risk (very low/low/intermediate) and higher risk (high/very high), ZBTB38 was significantly reduced in higher-risk MDS (0.99 [2.65–0.04]) in comparison to healthy donors, *P* < 0.05 (Fig. 6a). These results are in agreement with previously published microarray datasets from patients with MDS and AML^46^, that showed that *ZBTB38* mRNA is down-regulated in AML compared to control individuals (Supplementary Fig. S6A). *CDKN1C* mRNA expression did not significantly differ between patients with AML or MDS and healthy donors (Fig. 6b). It is possible that the expression of ZBTB38 is altered during cell differentiation and that its reduction in AML reflects the increased number of blasts. Nevertheless, our data suggest that the decrease in ZBTB38 expression is a possible marker of AML and high-risk MDS.

We further accessed the association between *ZBTB38* or *CDKN1C* mRNAs levels and the overall survival and event free survival of MDS patients in our cohort (most of them were not receiving hypomethylating agents). With an average follow-up of 30 months, we found that *ZBTB38* and *CDKN1C* mRNA levels do not independently predict overall survival or event free survival in MDS (Supplementary Table 1). IPSS-R was an independent prognostic factor for worse overall survival (HR 3.82 [95% CI, 1.5–9.4], *P* = 0.004), validating our cohort (Supplementary Table 1). Kaplan–Meier analysis indicated a poorer survival of MDS patients with lower ZBTB38 expression (below the median) in comparison with higher ZBTB38 (above the median), although not statistically significant (*P* = 0.054) (Fig. 6c). Overall, ZBTB38 mRNA expression is lower in higher-risk MDS patients and AML patient compared to healthy donors but does not predict overall survival and event free survival in MDS patients.

We then directly investigated the relationship between *RBBP6*, *USP9X*, *ZBTB38*, and *CDKN1C* expression and the clinical response to azacytidine in cancer patients. We retrieved the data reported for seven patients with MDS treated with 5-azacytidine plus entinostat (a potent HDACi)^1^. Three patients did not respond to chemotherapy, three patients had a partial clinical response and one patient presented a complete response. For each patient we have access to gene expression in the bone marrow prior to and 15 days after chemotherapy began. We observed no significant differences in the level of *ZBTB38, RBBP6*, and *USP9X* mRNA expression in samples from responder and non-responder patients at day 0 and at day 15 (Fig. 6d). In addition, we did not observe significant changes in the *ZBTB38*, *RBBP6*, and *USP9X* mRNA levels between day 0 and day 15 of treatment (Fig. 6d). On the contrary, we observed that *CDKN1C* mRNA expression was significantly higher in samples from responders compared to samples from non-responders at screening, day 0 (Fig. 6d). No clear difference is observed if the analysis is reproduced at day 15 post treatment (Fig. 6d). We propose that *CDKN1C* mRNA expression in MDS might be used to identify DNMTi responders but is not well suited to monitor drug response. Nonetheless, the small sample size of this study precludes a definitive conclusion on the predictive power of *CDKN1C* mRNA expression. Of note, we still observed the same trend when we extended the analysis to AML and CMML patients (4 extra-individuals) (Supplementary Figure S6B).

## Discussion

### ZBTB38 protein abundance is decreased by DNMTi

Cancer cells generally present global and local alteration in the pattern of DNA methylation^1,3,18,23^. In that framework, it has been proposed that DNMTi and other modulators of DNA methylation may have beneficiary effect in cancer treatments. In this study we found that 5-azacytidine and its derivatives cause the down regulation of ZBTB38 protein level without significantly affecting ZBTB38 mRNA expression or the expression of its paralogs ZBTB4 and ZBTB33/KAISO.

Decitabine, azacytidine, and zebularine are viewed as different types of DNMT inhibitors due to their structure, their mode of incorporation into the DNA as well as their metabolic fate^2,3,22,23^. These observations indicate that ZBTB38 degradation is most likely due to an indirect consequence of DNMTi cytotoxicity. We showed that ZBTB38 expression is not perturbed by treatments with siRNAs against DNMT1 or in HCT116 DKO cells that present different levels of DNA methylation loss; and that caffeine treatments dampens ZBTB38 downregulation upon azacytidine treatment, consistent with the involvment of a damage-induced signalisation in ZBTB38 regulation.

We also provide evidence that ZBTB38 poly-ubiquitination is involved in the control of ZBTB38 stability upon DNMTi treatment. While deubiquitinase USP9X is not mys-regulated upon azacytidine treatment in HeLa cells, E3 ubiquitin ligase RBBP6 is upregulated in DNMTi treated cells^31^. RBBP6 is involved in the maturation of messenger RNAs (mRNA) and controls the stability of a number of mRNAs in human cells^47^. This function of RBBP6 implicates structural domains present within the N-terminal portion of the protein and shared between different RBBP6 isoforms^47^. It is thus possible that DNMTi, by causing mRNA and DNA adducts and/or demethylation alters the expression, stability and/or function of RBBP6 isoforms and, in turn, contribute to the down-regulation of ZBTB38 protein expression.

### Implication of ZBTB38 in the toxicity of 5-azacytidine and its derivatives

Many models have been proposed to explain the toxicity of 5-azacytidine in cancer cells. Here, we demonstrate that transient inhibition of *ZBTB38* expression by siRNAs enhances the toxicity of 5-azacytidine, and its derivatives, in different cancer types. The consequences are dependent on the cell type owing to the presence of additional alterations affecting the sensibility to DNMTi (for example *TP53)*.

Our investigations indicate that depletion of *ZBTB38* does not enhance the demethylating activity of 5-azacytidine or the conversion of 5-methyl-cytosine into 5-hydroxy-methyl-cytosine. Consistent with this observation, we found that the genes encoding *DNMT1*, *TET2*, or *IDH1/2* enzymes were not regulated by *ZBTB38*.

We identified a recurrent up-regulation of *CDKN1C* expression at the mRNA level in different cancer cell types silenced for ZBTB38 or its deubiquitinase USP9X. However, when we studied the expression of *CDKN1C* at the protein level, we observed either a down-regulation or an upregulation in ZBTB38 silenced cells. A likely explanation is that *ZBTB38* down-regulation disrupts *CDKN1C* regulation transcriptionally and post-translationally in presence of DNMTi. The analysis of ZBTB38 genomic targets by chromatin immunoprecipitation does not identify binding sites for ZBTB38 in the ICR2 region nor at the *CDKN1C* promoter in HeLa cells (C.M., P.A.D. and B.M., unpublished data). The regulation is thus likely to be indirect or the recruitment of ZBTB38 highly dynamic at the promoter of *CDKN1C*.

The 11p15.5 region containing *CDKN1C* is associated with growth disorders such as the Silver–Russell syndrome and the Beckwith–Wiedemann syndrome^42,48^. Intriguingly many polymorphisms in the *ZBTB38* locus are linked to the regulation of human height and to idiopathic growth syndrome and some of these polymorphisms affect *ZBTB38* expression level^49,50^. It is thus tempting to speculate that *ZBTB38* might regulate the expression of *CDKN1C* in pathological but also physiological context including during embryonic development. Further studies will be required to clarify the direct and indirect regulation of *CDKN1C* expression by *ZBTB38* in normal and cancer cells. It is also likely that additional targets and pathways are mys-regulated upon ZBTB38 depletion and that they may also contribute to ZBTB38 effects, explaining variable effects on CDKN1C protein levels and DNMTi toxicity in different cell types.

### Can ZBTB38 inactivation further enhance the efficacy of DNMTi inhibitors in clinic?

Prediction of cancer drug response is an important challenge^8,51,52^. Markers able to predict the response to DNMTs inhibitors and individuals most likely to benefit from the therapy have been largely unsuccessful and not yet validated in clinic^1,9,21,51–53^.

To test the possibility that USP9X, RBBP6, ZBTB38, and/or CDKN1C could be predictive markers, we retrieved the clinical and gene expression data from a previously published cohort of patients with MDS showing effective and tolerable effects of 5-azacytidine^1^. Using this information on seven patients, we observed a correlation between a high level of *CDKN1C* mRNA expression before treatment and the clinical response to 5-azacytidine in patients with MDS. In haematopoietic cells derived from MDS and AML patients the *CDKN1C* promoter is mostly unmethylated^1^. The baseline level of *CDKN1C* mRNA expression prior treatment might thus help the identification of patients could benefit from a chemotherapy based on a combination of DNMTi and HDACi. In addition, enhancing *CDKN1C* mRNA basal expression by disrupting ZBTB38 or USP9X might help further enhance the response to DNMTi in clinic or define new chemotherapeutic cocktails.

The analysis of our own cohort of patients, that for most of them were not receiving hypomethylating agents, indicate that *ZBTB38* and *CDKN1C* mRNA levels do not predict overall survival in these patients with MDS. Previous works indicate that CDKN1C protein expression scored by immunohistochemistry (but not mRNA expression) prior to treatment help anticipate the clinical response of patients with MDS to conventional chemotherapy^37^. These data suggest that monitoring both *CDKN1C* mRNA and protein expression levels in patients might help classify potential responders to 5-azacytidine treatment or conventional care therapy. Our study grants the analysis of a larger number of patients with MDS/AML under hypomethylating therapy or conventional care regimens to further support the observation that *CDKN1C* mRNA might help predict 5-azacytidine efficacy. Increasing the number of patients will also allow the consideration of genomic abnormalities and clinical features in patients that could mask or unravel CDKN1C predictive value, including the status of tumor suppressor TP53 and DNMTs. The status of *CDKN1C* expression could also be tested in other cancers as 5-azacytidine plus entinostat regimen has been successfully tested on patients with AML arising after chemotherapy or radiation therapy, breast cancer and colorectal cancer^5,7^.

Our work provides a better understanding of 5-azacytidine action in cancer cells and it might help improve current predictive strategies for determining good outcome and survival upon 5-azacytidine-based therapy in cancer.

## Materials and methods

### Cell culture conditions

The cells were grown in a humidified atmosphere of 5% CO_2_ at 37 °C and the media changed every 2 days. A detailed description of culture condition and media composition is provided in the supplementary section.

### Chemicals and siRNAs

5-Azacytidine (Sigma-Aldrich; A1287), 5-Aza-2’-Deoxycytidine/Decitabine (Sigma-Aldrich; A3656) and Pyrimidin-2-one Beta-Ribofuranoside/Zebularine (Sigma-Aldrich; Z4775) were freshly resuspended in DMSO at the concentration recommended by supplier. The suspension was further diluted in the culture media for the experiment to final working concentration of up to 10 µM. Cells were treated with media containing 5-azacytidine (or derivatives) replaced every 24 h to ensure proper action of the drug(s) for long-term expositions. Proteasome inhibitor MG132 was purchased from Merck Millipore (InSolution^TM^ MG132) and it was used at final concentration of 10 µM for 4 h. Caffeine was purchased from Sigma-Aldrich (C0750) and it was used at final concentration of 1 mM for 24 h.

Control scrambled siRNAs and siRNAs directed against ZBTB38, USP9X, p53, CDKN1C and DNMT1 were purchased from Thermo Fisher Scientific and some were validated in previous studies^31,32^. siRNAs were delivered in adherent cells using Lipofectamine 3000 following protocols provided by the manufacturer (Thermo Fisher Scientific). Cells in suspension (K562, MOLM-14 and THP-1) were electroporated using the Neon^®^ transfection system (Thermo Fisher Scientific) using the kit MPK10096 and the following parameters: pulse voltage at 1450 V, pulse width 10 milliseconds and a total of 3 sequential pulses.

### Colony forming assays

A fixed number of adherent cells (200 cells) were seeded in 6-well plates. Typically, each plate is prepared in triplicate to control plating efficiency and drug efficiency. After 24 h in the incubator the cells were exposed to different concentration of 5-azacytidine (or derivatives) for 48 h. The cells were then incubated in fresh media for up to 15 days (depending on the cell type). The cells were fixed, under a fume hood, with formaldehyde, stained with crystal violet and extensively washed with distilled water. The number of colonies per well was then manually counted and also access using the Image J software on scanned images of the plates. Concordant results were further considered, and the ratio between the number of colonies in the treated condition normalized to the number of colonies in the control condition reported in graphs.

### Cell proliferation assay with suspension cells

K562, MOLM-14 and THP-1 cells were seeded in 6-well plate and treated with adequate concentrations of DNMTi. At the different time points the number of cells per well was manually monitored using a KOVA slide with counting grid. Each experiment was technically replicated and we performed at least three independent biological replicate. The condition without DNMTi was used as a control and all data in each experiment were expressed relative to its value (set up to 1). In the case of proliferation curve analysis the data are expressed relative to the number of cells at day 0 (day of DNMTi removal) in each condition.

### GEO datasets and bioinformatics analysis

The different datasets analyzed in this study were retrieved from the NCBI gene expression omnibus portal from previously published work and are detailed in the supplementary section.

Correlation between gene expression levels in multiple conditions was accessed by Pearson’s coefficient analysis and presented as the square of the Pearson’s coefficient in graphs. Comparison of mean ranks was accessed by Mann–Whitney *U*-test and the *p*-value reported.

### DNA methylation analysis

Global and site-specific analysis of DNA methylation were conducted using the LUminometric-based Methylation Assay (LUMA), dot blots and methylated DNA immunoprecipitation (MeDIP) analysis using previously described protocols. The complete methodologies are provided in the supplementary section.

### Western blot analysis; Gene expression analysis; Flow-cytometry analyses; Bio-informatic analysis

Methods are presented in details in the supplementary section.

## Electronic supplementary material

Supplemental Material
