# Supplementary material for: Depletion of ZBTB38 potentiates the effects of DNA demethylating agents in cancer cells via CDKN1C mRNA up-regulation
Source: Oncogenesis. 2018 Oct 11;7(10):82. doi: 10.1038/s41389-018-0092-0 (PMC6182000; doi:10.1038/s41389-018-0092-0)
Supplement: Supplementary file 1 — Supplemental Material [file 41389_2018_92_MOESM1_ESM.pdf]

SUPPLEMENTARY MATERIAL

Depletion of *ZBTB38* potentiates the effects of DNA demethylating agents in cancer cells via *CDKN1C* mRNA up-regulation

Claire Marchal, Maud de Dieuleveult, Claude Saint-Ruf, Nadège Guinot, Laure Ferry, Sara T. Olalla Saad, Mariana Lazarini, Pierre-Antoine Defossez and Benoit Miotto

The file contains:

- Legends to Supplementary Figures S1 to S5.
- Supplementary Figures S1 to S5.
- Supplementary Materials and Methods.
- Bibliographic References.
- Supplementary Table 1 to 5.

Legend to supplementary figures:

Supplementary Figure S1:

ZBTB38 degradation upon 5-azacytidine exposure is influenced by the genetic status of tumor suppressor *TP53* and DNA methylases *DNMT1* and *DNMT3A*; and it coincides with E3 ligase RBBP6 up-regulation. (**A**) Gene expression analysis of *ZBTB38* by real-time PCR in U2OS, HepG2 and HCT116 cells treated with 5-azacytidine (Aza) compared to control cells (n=3, same samples as in Figure 1F). Expression level is expressed as the fold change between treated and control cells. (**B**) Gene expression analysis of *ZBTB38* by real-time PCR in THP-1 and MOLM-14 cells treated with 5-azacytidine (Aza) compared to control cells (n=3, same samples as in Figure 1G). Expression level is expressed as the fold change between treated and control cells. (**C**) Western blot analysis of ZBTB38, DNMT1 and GAPDH protein levels in HCT116, HCT116 *DKO* (*DNMT1* and *DNMT3A* knockout) and HCT116 $p53^{-/-}$ cells exposed to increasing concentration of 5-azacytidine (Aza): 0, 2 and 10 μM. (**D**) Quantification of western blots presented in panel S1C using the ImageJ software. Intensity values are normalized to condition without 5-azacytidine set up as 1. (**E**) Western blot analysis of ZBTB38, P53 and GAPDH protein levels in MOLM-14 cells transfected with siRNA control and against p53 and further exposed to increasing concentration of 5-azacytidine (Aza): 0, 2 and 10 μM. (**F**) Western blot analysis of RBBP6 protein expression in HeLa cells treated with 5-azacytidine (Aza) compared to control (Ctrl) cells (same sample as in Figure 1D). (**G**) Gene expression analysis of *RBBP6* by real-time PCR in HeLa cells treated with 5-azacytidine (Aza) compared to control cells (n=3; same samples as in Figure 1E). Expression level is expressed as the fold change between treated and control cells. Asterisk (*) indicates a significant difference in between control and treated cells (t-test, P-value $P<0.01$). (**H**) Western blot analysis of USP9X protein expression in HeLa cells treated with 5-azacytidine (Aza) compared to control (Ctrl) cells. (**I**) Graph representing the relative methylation level of the CpG island promoter of *RBBP6*, *ZBTB38*, *MCM10* compared to two hyper-methylated CpG islands located in the promoter of *VASH2* and *CDH13* in HeLa cells. Methylation at individual loci was normalized to a genomic region CpG-free set up as 1 (n=3). (**J**) RT-PCR analysis of *ZBTB38* mRNA expression in HeLa and HCT116 cells treated with 10 μM of either 5-azacytidine, decitabine or zebularine and control mock-treated cells. The expression value in mock treated cells is artificially set up as 1 (n=3; same samples as in Figure 1J). **(K)** Quantification of the level of global DNA methylation using a luminometric-methylation assay (LUMA) in colon HCT116 cells double knock-out for

DNMT1 and *DNMT3B* (*DKO*) and their isogenic counterpart (*WT*) (n=3). (**L**) Western blot analysis of ZBTB38, DNMT1 and GAPDH protein levels in HCT116 *DKO* cells compared to isogenic cells.

<u>**Supplementary Figure S2:**</u>

Depletion of *DNMT1* by RNA interference does not affect ZBTB38 protein expression level while reactivating tumor suppressor gene expression. Expression of control and heavily-methylated tumor suppressor genes in samples analyzed in panel K of Figure 1. We monitored the expression of *CDH1* in HeLa cells, *MAGE10A* and *DCSR8* in HCT116 cells and *MYT1* in U2OS cells. These genes were previously shown to be reactivated upon DNA demethylation (Viré et al., 2006; Leung et al., 2008; Clements et al., 2012).

<u>**Supplementary Figure S3:**</u>

Inactivation of *ZBTB38* by RNA interference increases decitabine and zebularine cytotoxicity in a panel of cancer cells. (**A**) Depletion of *ZBTB38* enhances the cytotoxicity of decitabine in HeLa, U2OS, K562, MOLM-14 and THP-1 cells (n=3). ***, $P<0.01$. *, $P<0.05$. (**B**) Depletion of *ZBTB38* enhances the cytotoxicity of zebularine in HeLa and U2OS cells (n=5). ***, $P<0.001$. (**C**) Graph representing the number of colonies when *ZBTB38* depletion is performed prior or after decitabine exposure. Left panel: data on HeLa cells. Right panel: data on U2OS cells. The bars represent the ratio of colonies in the condition siRNA *ZBTB38* versus siRNA Control. A score of 1 indicate no significant effect on cell viability (n=4). (**D**) Representative images of wells analyzed in panel S3C. (**E**) Western blot analysis of ZBTB38 expression level in the two experimental set up confirmed efficient knock-down. The cellular extracts were prepared 48 hours after transfection of the siRNA molecules.

<u>**Supplementary Figure S4:**</u>

Inactivation of *USP9X* or *ZBTB38* by RNA interference causes a strong arrest in cell proliferation upon DNMTi exposure, persistant after removal of DNMTi. (**A**) Graph depicting the growth curve of THP-1 cells transfected with siRNAs (control and USP9X) and with decitabine 1 μM (DAC) or not (n=4). ***, P<0.001. Right panel: Western blot analysis of ZBTB38 and USP9X expression in the different conditions. (**B**) same experiment in MOLM-14 cells. Right panel: Western blot analysis of ZBTB38 and USP9X expression in the different conditions. (**C**) Graph depicting the growth curve of THP-1 (left) and MOLM-14 (right) cells transfected with siRNAs (control, ZBTB38 and USP9X) and with decitabine 0.1 μM (DAC)

for 5 days (n=4). ***, P<0.001.

<u>Supplementary Figure S5:</u>

Inactivation of *ZBTB38* in THP-1 and HeLa slightly enhances cell death in presence of decitabine. (**A**) Representative FACS analysis of autophagy in THP-1 cells after treatment with *ZBTB38* and control siRNAs and further exposed to decitabine or not. (**B**) Quantification of autophagic cells (gate C in panel S5A) in THP-1 and HeLa cells transfected with *ZBTB38* and control siRNAs and further exposed to DNMT inhibitors or not. (**C**) Western blot analysis of autophagic markers P62/SQSTM1 and LC3I-II expression in HeLa cells transfected with ZBTB38 and control siRNAs and further exposed to decitabine or not. (**D**) Representative FACS analysis of cell cycle phases in THP-1 cells after treatment with *ZBTB38* and control siRNAs and further exposed to decitabine or not. (**E**) Quantification of G1, S and G2/M cells in THP1 and HeLa cells transfected with *ZBTB38* and control siRNAs and further exposed to DNMT inhibitors or not.

(**F**) Representative FACS analysis of apoptosis and necrosis in THP-1 cells after treatment with *ZBTB38* and control siRNAs and further exposed to decitabine or not. (**G**) Quantification of late apoptotic (black bar), early apoptotic (grey bar) and necrotic (light grey bar) cells in THP-1 and HeLa cells transfected with *ZBTB38* and control siRNAs and further exposed to DNMT inhibitors or not (DAC: decitabine 1μM, 24 hours; AZA: azacytidine 4μM, 24 hours). (**H**) Quantification of cell debris in THP-1 and HeLa cells transfected with *ZBTB38* and control siRNAs and further exposed to DNMT inhibitors or not (similar condition as panel S6B).

<u>Supplementary Figure S6:</u>

ZBTB38 and CDKN1C mRNA expression in patients with MDS and AML. (**A**) Relative expression level of *ZBTB38* and *CDKN1C* mRNAs in the bone marrow of patients with MDS and AML as well as healthy individuals (data from Haferlach et al., 2010). *P*-value between the different groups (patients versus control) were calculated using the Mann-Whitney U-test. (**B**) Expression of *CDKN1C* mRNA prior to DNMTi treatment in samples from patients with MDS (left panel) and in patients with MDS, AML or CMML (right panel). Responders to the therapy are highlighted in orange and non-responders presented in grey (expression and clinical data from Fandy et al., 2009).

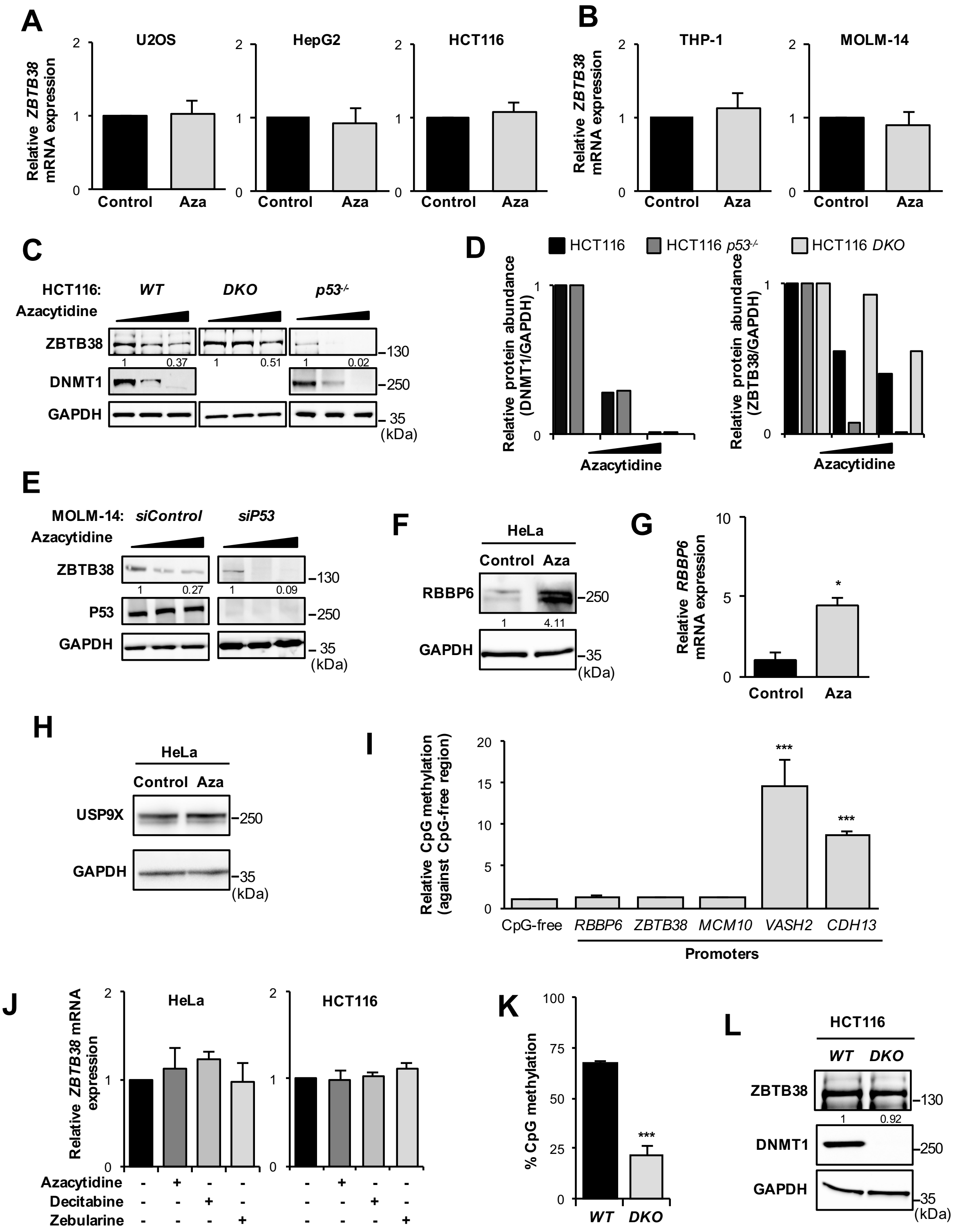

Supplementary Figure S1 :

ZBTB38 degradation upon 5-azacytidine exposure is influenced by the genetic status of tumor suppressor *TP53* and DNA methylases *DNMT1* and *DNMT3A*; and it coincides with E3 ligase RBBP6 up-regulation.

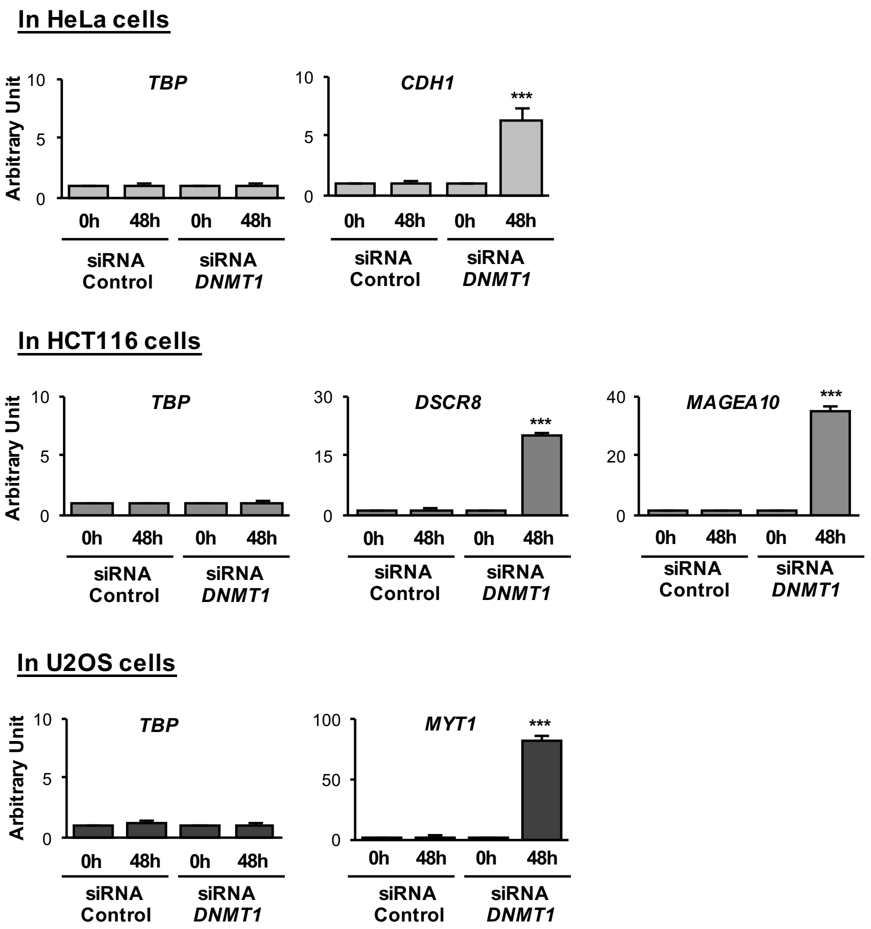

Supplementary Figure S2 :
Depletion of *DNMT1* by RNA interference does not affect ZBTB38 protein expression level while reactivating tumor suppressor gene expression.

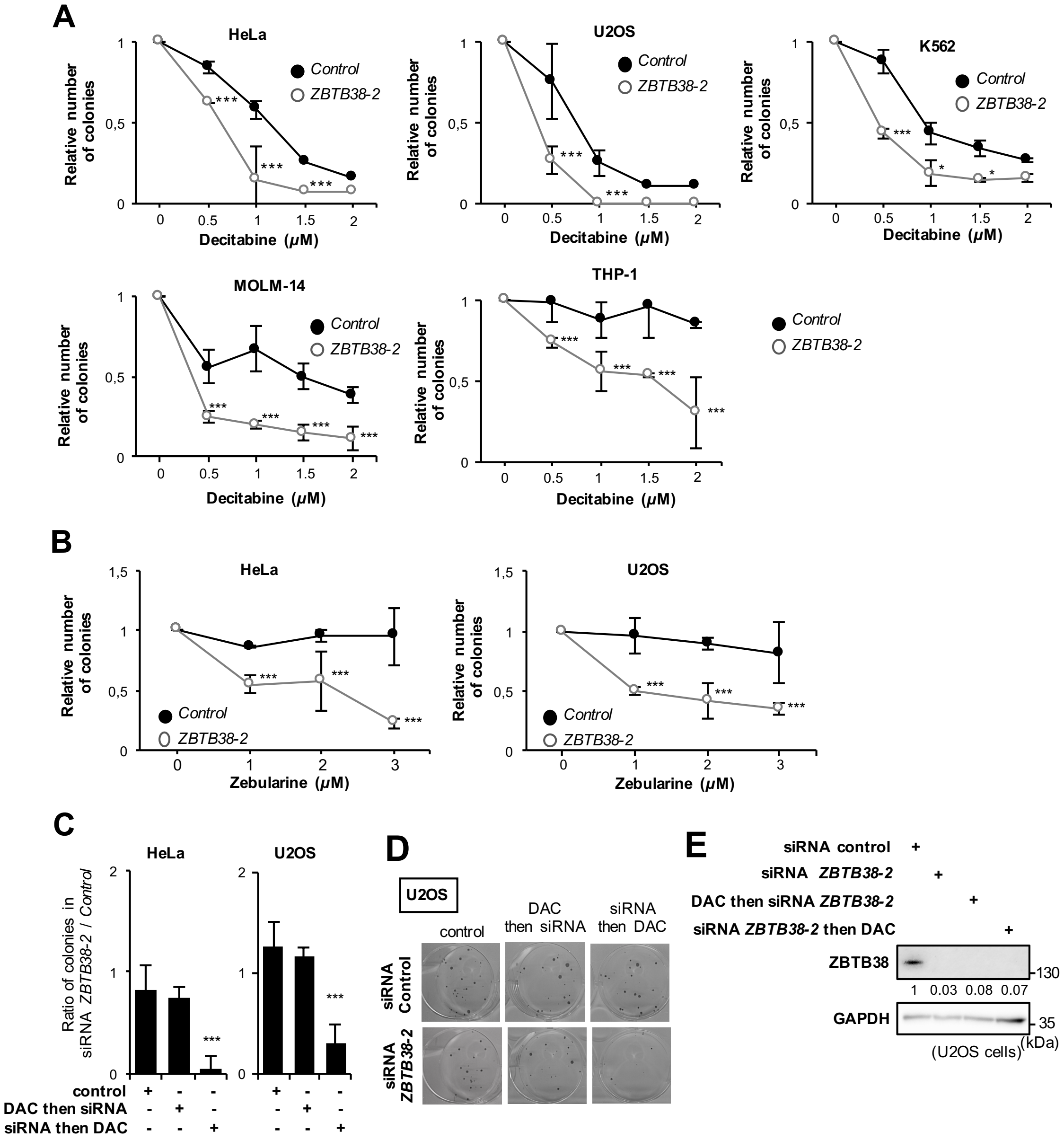

Supplementary Figure S3:

Inactivation of *ZBTB38* by RNA interference increases decitabine and zebularine cytotoxicity in a panel of cancer cells

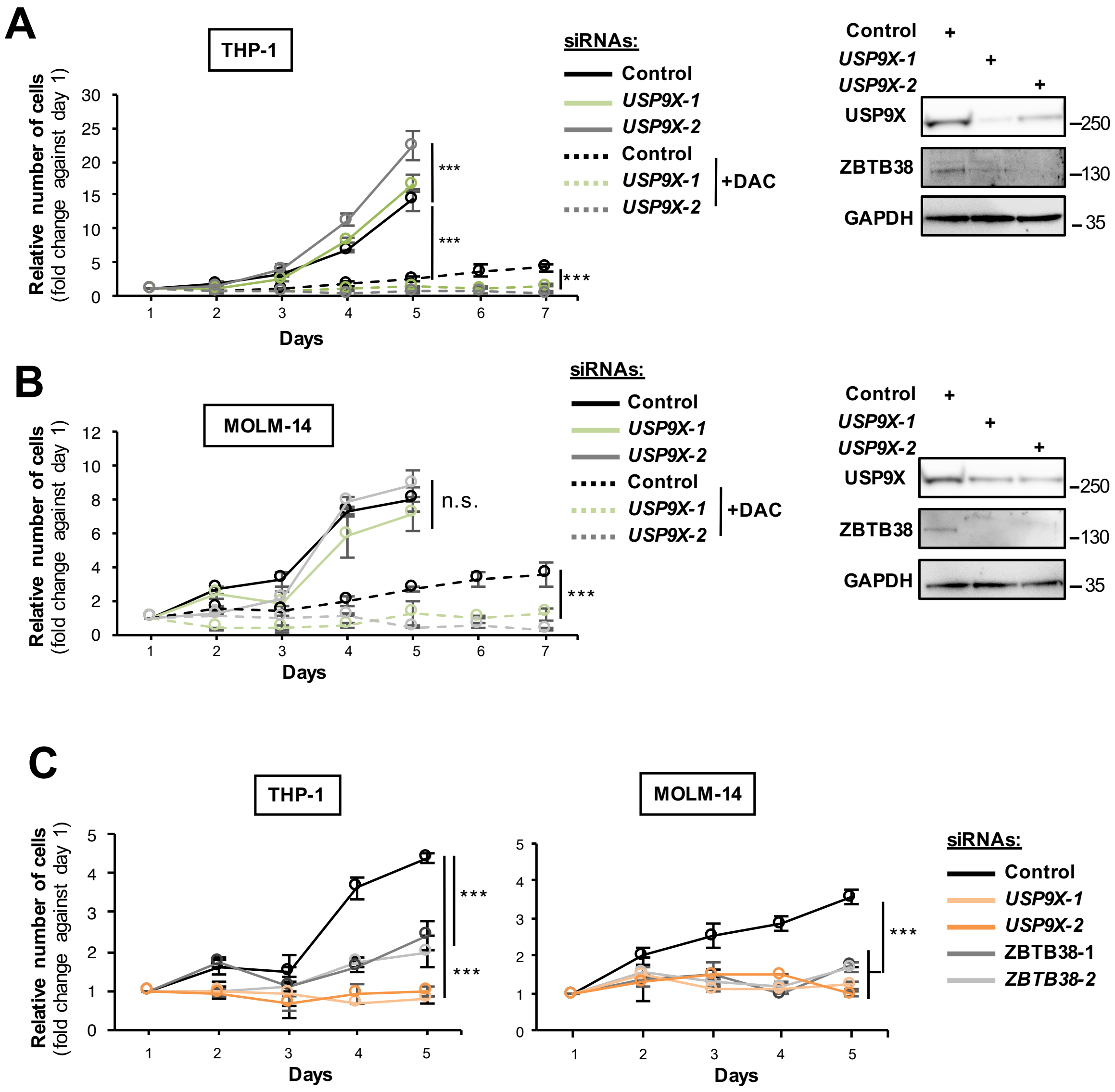

Supplementary Figure S4:

Inactivation of *USP9X* or *ZBTB38* by RNA interference causes a strong arrest in cell proliferation upon DNMTi exposure, persistant after removal of DNMTi.

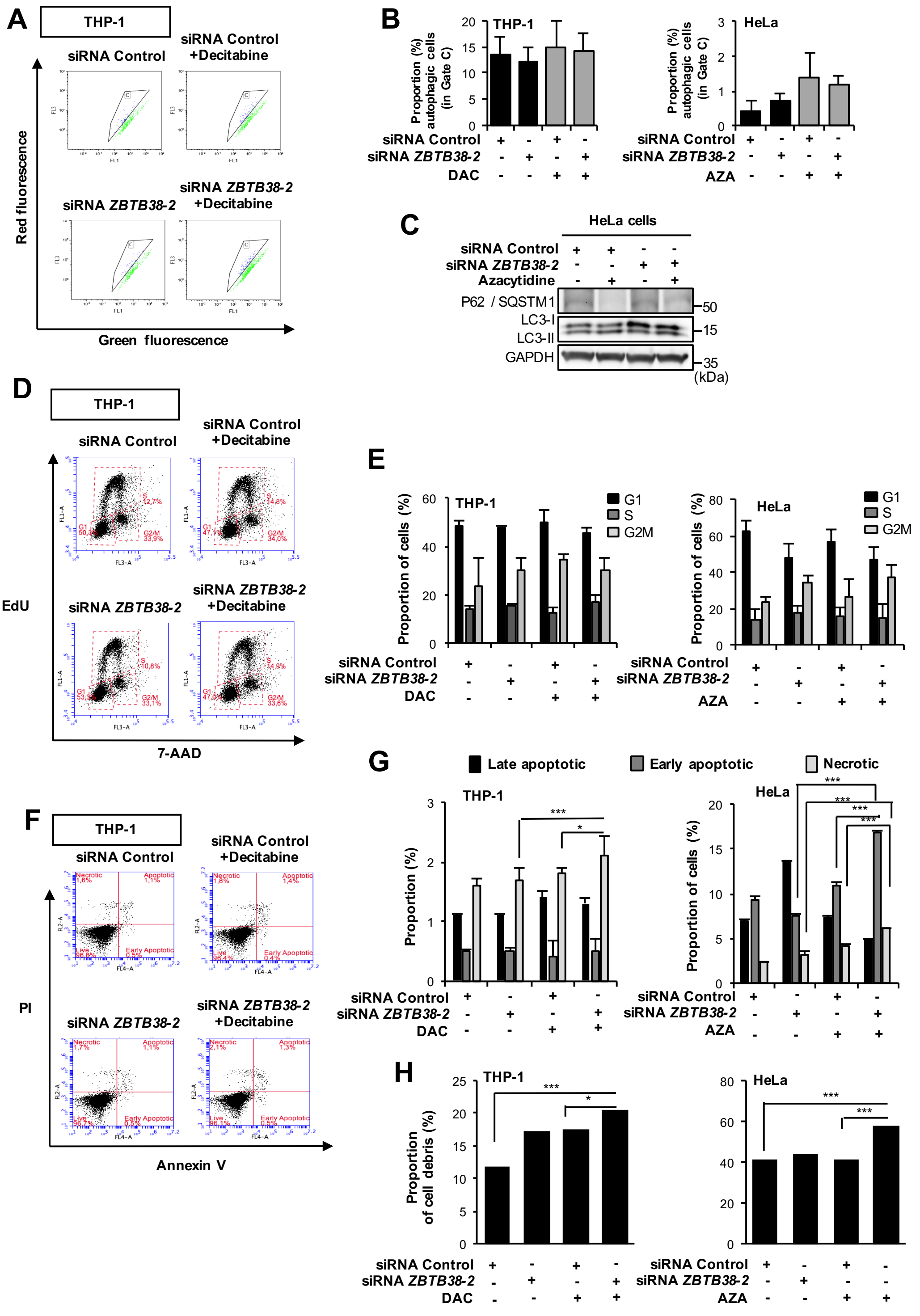

Supplementary Figure S5:
Inactivation of *ZBTB38* in THP-1 and HeLa cells slightly enhances cell death in presence of decitabine.

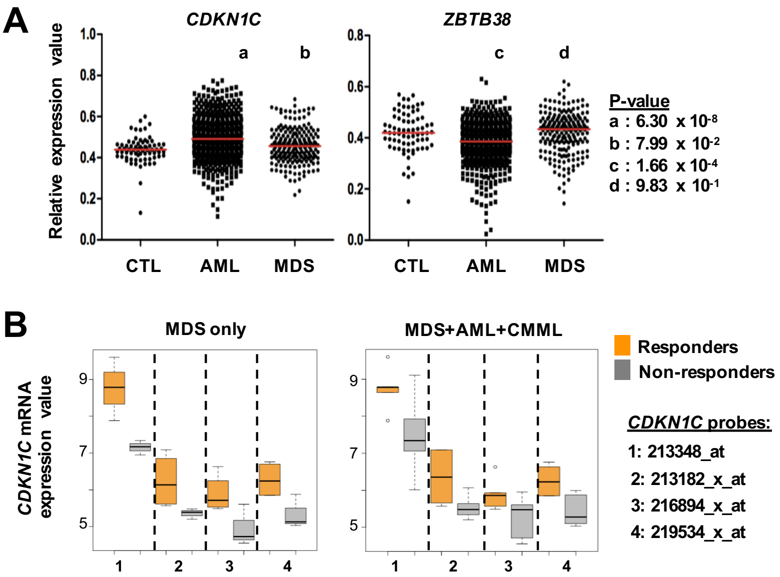

Supplementary Figure S6:

ZBTB38 **and** ***CDKN1C*** **mRNA expression in patients with MDS, CMML and AML treated with azacytidine.**

Supplementary Materials and Methods:

Cell culture conditions.

The cells were grown in a humidified atmosphere of 5% CO2 at 37°C and the media change every 2 days. The colon cancer HCT116 (*p53*$^{+/+}$), HCT116 *DNMT1*$^{-/-}$ *DNMT3B*$^{-/-}$ (DKO) and HCT116 (*p53*$^{-/-}$) cells were cultured in McCoy's 5A modified media (Thermo Fisher Scientific) supplemented with 10% fetal bovine serum (Gibco® sera, South America origin). The human U2OS, HepG2, DU145 and HeLa cells were cultured in Dulbecco's modified Eagle's medium (DMEM) (Thermo Fisher Scientific) supplemented with 10% fetal bovine serum (Gibco® sera, South America origin). The THP-1, MOLM-14 and K562 cells were cultured in RPMI 1640 (Thermo Fisher Scientific) medium supplemented with 10% fetal bovine serum (Gibco® sera, South America origin).

HeLa, U2OS, HCT116, K562 and MOLM-14 were in our lab stocks and their genotypes were confirmed by cell authentication (Eurofins) (certificates available in Supplementary Table 5). Other cells were directly obtained from colleagues. HCT116 (*p53*$^{+/+}$), HCT116 *DNMT1*$^{-/-}$ *DNMT3B*$^{-/-}$ (DKO) and HCT116 (*p53*$^{-/-}$) cells were kindly provided by Dr. B. Vogelstein (Bunz et al., 1998: Rhee et al., 2002). HepG2 and THP-1 cells were obtained by courtesy of Dr. Jean-Pierre Couty and Dr. Isabelle Dusanter respectively (Institut Cochin, INSERM U1016, Paris). The lack of *TP53* expression in THP-1 cells was confirmed by western blot (Figure 5A).

Western blot analysis.

Cell extracts were prepared as previously described in RIPA buffer supplemented with protease and phosphatase inhibitors (Miotto et al., 2014), resolved on Bolt pre-cast gels (Invitrogen) and then transfered to Immobilon-P membranes (Millipore). The membranes were blocked with 5% fat-free milk in PBS, then incubated overnight at 4 °C with the appropriate primary antibodies. The membranes were incubated with the cognate secondary antibody coupled to horseradish peroxidase, and revealed using the West Dura kit (ThermoFisher Scientific), in the ChemiSmart 5000 imager (Vilber Lourmat). References of the primary antibodies are provided in Supplementary Table S2. Secondary antibodies coupled with horseradish peroxidase were purchased from Jackson ImmunoResearch.

Digital images were used for semi-quantification of protein expression on the Image J software using GAPDH as the reference.

Gene expression analysis.

mRNAs were prepared using TriReagent protocol (Sigma-Aldrich; T9424) and followed by a standard phenol-chloroform purification procedure. mRNAs were reverse-transcribed using the SuperScript II (or IV) reverse transcriptase following manufacturer procedures (Thermo Fischer Scientific). cDNAs were analyzed by real-time PCR on a LightCycler® 480 system (Roche) available at the GENOM'IC platform at Institut Cochin (INSERM U1016, Paris). The list of specific primer pairs is provided in Supplementary Table S3. Relative gene expression level were determined using the $2^{-\Delta\Delta Ct}$ method and the data normalized to the expression of a set of 3 housekeeping genes (*GAPDH*, *MAPK14* and *TFRC*) as previously described (Miotto et al., 2014).

Flow-cytometry analyses

Flow-cytometry based analysis were performed on a BD Accuri™ C6 (BD Biosciences) available at the CYBIO platform at Institut Cochin (INSERM U1016, Paris). All data were visualized, analyzed and processed on the BD Accuri™ C6 analysis software. Ten thousand to twenty thousand cells were analysis per condition.

Cell cycle analysis were performed using the Click-IT Edu assay kit for flow cytometry using protocols provided by the manufacturer (Thermo Fisher Scientific). Briefly, cells were grown in the presence of 5-ethynyl-2′-deoxyuridine for 40 minutes. Following fixation and Click-IT reaction cells were further stained with 7-aminoactinomycin D to detect total DNA content.

Cell death analysis were performed by co-staining cells with propidium iodide and Annexin V on fresh cells using the Annexin V apoptosis detection kit APC (eBiosciences) following the recommendations of the manufacturer.

Autophagy was monitored by visualizing the intensity of the acidic cellular compartment using acridine orange staining. Cells were incubated with medium containing 1 μg/mL acridine orange (Invitrogen A3568) for 20 minutes, washed once with PBS and the red and green fluorescence quantified by FACS.

GEO datasets and bioinformatics analysis

Expression data from MDS patients treated with a combination of 5-azacytidine and entinostat were retrieved from GSE16625 (Fandy et al., 2009). Patients enrolled in the study were treated with sequential administration of 5-azacytidine and entinostat. 5-azacytidine was administered subcutaneously for 10 consecutive days in doses of 30, 40 or 50 mg/m2 per day. Entinostat (2,

4, 6 or 8 mg/m2) was administered orally on days 3 and 10 of the 5-azacytidine treatment. CD34-positive samples were obtained before treatment (Day 0) and on day 15. The clinical response of each patient was assessed using International Working Group 2000 criteria and previously reported (Fandy et al., 2009).

Gene expression in the bone marrow of AML and MDS patients was re-analyzed from microarray data deposited as GSE13159 (Haferlach et al., 2010). The cohort comprised 72 control individuals, 542 patients with AML and 206 patients with MDS.

Gene expression data of developing human erythroid progenitors were re-analyzed from microarray data deposited as GSE4655, GSE100354 and GSE2666 (Keller et al., 2006; Eckfeldt et al., 2005).

Bone marrow samples and survival outcomes in MDS patients

Bone marrow samples were collected from patients with MDS (n=55), AML with myelodysplasia-related changes (AML-MRC) (n=16), *de novo* AML (n=47) and from healthy donors (n=12) at the Hematology and Blood Transfusion Center of the University of Campinas, Brazil. All patients were untreated at the time of sample collection and their characteristics are described in Supplementary Table S4. The study was conducted in accordance with the Declaration of Helsinki and was approved by the local Ethical Committee Board. Gene expression comparisons were performed with the Kruskall-Wallis test followed by Dunn's multiple comparison test. Cox regression model was used to estimate overall survival (OS) and event free survival (EFS) of MDS patients. OS was defined as the time between the date of sampling and the date of death (for deceased patients) or last follow up (for censored patients). EFS was defined as the time between the sampling and the date of the first event (death or MDS progression to a higher risk MDS category by the World Health Organization or to AML-MRC) or last follow up (for censored patients). *Multivariate analyses* were conducted using *univariate analysis* variables at a P level of <0.12. A *P* value ≤0.05 was considered as statistically significant.

DNA methylation analyses

LUminometric-based Methylation Assay (LUMA) was performed as previously described (Karimi et al., 2011). Briefly, purified genomic DNA were digested by two different mix of enzymes: HpaII+EcoRI or MspI+EcoRI (all purchased from New England Biolabs). The samples are then run on the pyro-sequencing platform (Pyromark Q24, Qiagen) located at UMR7216 'Epigenetic and Cell Fate' in Paris (www.parisepigenetics.com/ecf). The estimate

of the HpaII/MspI ratio (normalized to EcoRI in each sample) define the relative methylation level of the genomic DNA.

Dot blots were conducted using a specific antibody directed against 5-methyl-CpG (Active Motif, 61480) and 5-hydroxymethyl-CpG (Active Motif, 39769) and normalized to total DNA using an antibody directed against single-strand DNA (Millipore, MAB3034) in each samples. Genomic DNAs were heat-denaturated and an equal amount of DNA for each condition spotted on a nylon membrane (Sigma-Aldrich). Following incubation with antibodies and signal detection, intensity of 5(h)mC and total DNA signals were evaluated using Image J software and the ratio 5mC/DNA and 5hmC/DNA calculated.

MeDIP (Methylated DNA immunoprecipitation) analysis was conducted using the Auto-MeDIP kit (Diagenode) using the manufacturer conditions. Briefly, genomic DNA was purified from samples, sonicated to the range of 200 base pairs, denaturated by heat and immunoprecipitated using a methyl-CpG specific antibody (Monoclonal antibody 33D3; C15200081). Precipitated DNAs were then analyzed by real-time qPCR on an Applied Biosystems 7500 real-time machine using specific primers. The primers are as followed: MCM10 promoter 5'-AGG GAC CTT CCT GTC GGT AG-3' and 5'-ACT TAA ACT TCC CGG CAC CA-3'; VASH2 promoter 5'-GAG TTC CAG CGC CTA TCA CC-3' and 5'-GTC CCG AGG TAG GAT CTT GG-3'; CDH13 promoter 5'-TTG GGT AGA GGC TGA TGA CC-3' and 5'-TAT CTG CCA TGC AAA ACG AG-3'; CpG-free region 5'-CTG AAT CAG CAG ACA GAA TGG A-3' and 5'-GGT AGG CAA CAC AGG TTT GG-3'; RBBP6 promoter 5'-ATC CAC GGA GAG AGA GAC CC-3' and 5'-GAT TGG CGG TGC AGT CAG TA-3'; ZBTB38 promoter 5'-TCT GCG AAT CCA GTG TGC AT-3' and 5'-AGT TAG GAC TCT GGG CCA CA-3'.

Bibliographic references:

Bunz F, Dutriaux A, Lengauer C, Waldman T, Zhou S, Brown JP, Sedivy JM, Kinzler KW, Vogelstein B. Requirement for p53 and p21 to sustain G2 arrest after DNA damage. Science. 1998 Nov 20;282(5393):1497-501.

Clements EG, Mohammad HP, Leadem BR, Easwaran H, Cai Y, Van Neste L, Baylin SB. DNMT1 modulates gene expression without its catalytic activity partially through its interactions with histone-modifying enzymes. Nucleic Acids Res. 2012 May;40(10):4334-46.

Eckfeldt CE, Mendenhall EM, Flynn CM, Wang TF, Pickart MA, Grindle SM, Ekker SC, Verfaillie CM. Functional analysis of human hematopoietic stem cell gene expression using zebrafish. PLoS Biol. 2005 Aug;3(8):e254.

Fandy TE, Herman JG, Kerns P, Jiemjit A, Sugar EA, Choi SH, Yang AS, Aucott T, Dauses T, Odchimar-Reissig R, Licht J, McConnell MJ, Nasrallah C, Kim MK, Zhang W, Sun Y,

Murgo A, Espinoza-Delgado I, Oteiza K, Owoeye I, Silverman LR, Gore SD, Carraway HE. Early epigenetic changes and DNA damage do not predict clinical response in an overlapping schedule of 5-azacytidine and entinostat in patients with myeloid malignancies. Blood. 2009 Sep 24;114(13):2764-73.

Haferlach T, Kohlmann A, Wieczorek L, Basso G, Kronnie GT, Béné MC, De Vos J, Hernández JM, Hofmann WK, Mills KI, Gilkes A, Chiaretti S, Shurtleff SA, Kipps TJ, Rassenti LZ, Yeoh AE, Papenhausen PR, Liu WM, Williams PM, Foà R. Clinical utility of microarray-based gene expression profiling in the diagnosis and subclassification of leukemia: report from the International Microarray Innovations in Leukemia Study Group. J Clin Oncol. 2010 May 20;28(15):2529-37.

Karimi M, Luttropp K, Ekström TJ. Global DNA methylation analysis using the Luminometric Methylation Assay. Methods Mol Biol. 2011;791:135-44.

Keller MA, Addya S, Vadigepalli R, Banini B, Delgrosso K, Huang H, Surrey S. Transcriptional regulatory network analysis of developing human erythroid progenitors reveals patterns of coregulation and potential transcriptional regulators. Physiol Genomics. 2006 Dec 13;28(1):114-28.

Leung RC, Liu SS, Chan KY, Tam KF, Chan KL, Wong LC, Ngan HY. Promoter methylation of death-associated protein kinase and its role in irradiation response in cervical cancer. Oncol Rep. 2008 May;19(5):1339-45.

Miotto B, Chibi M, Xie P, Koundrioukoff S, Moolman-Smook H, Pugh D, Debatisse M, He F, Zhang L, Defossez PA. The RBBP6/ZBTB38/MCM10 axis regulates DNA replication and common fragile site stability. Cell Rep. 2014 Apr 24;7(2):575-87.

Rhee I, Bachman KE, Park BH, Jair KW, Yen RW, Schuebel KE, Cui H, Feinberg AP, Lengauer C, Kinzler KW, Baylin SB, Vogelstein B. DNMT1 and DNMT3b cooperate to silence genes in human cancer cells. Nature. 2002 Apr 4;416(6880):552-6.

Viré E, Brenner C, Deplus R, Blanchon L, Fraga M, Didelot C, Morey L, Van Eynde A, Bernard D, Vanderwinden JM, Bollen M, Esteller M, Di Croce L, de Launoit Y, Fuks F. The Polycomb group protein EZH2 directly controls DNA methylation. Nature. 2006 Feb 16;439(7078):871-4.

Supplementary Table 1:

Univariate and Multivariate analyses of survival outcomes in MDS patients

	Univariate analysis						Multivariate analysis					
	Event-Free Survival			Overall Survival			Event-Free Survival			Overall Survival		
**Factor**	**HR**[1]	**(95% C.I.)**	***p***	**HR**	**(95% C.I.)**	***p***	**HR**	**(95% C.I.)**	***p***	**HR**	**(95% C.I.)**	***p***
**Gender**												
Male vs female	1.45	0.70 - 3.02	0.321	1.39	0.63 - 3.07	0.410	-	-	-	-	-	-
**Age at sampling**												
≥ 60 *vs.* <60	1.07	0.47 – 2.43	0.868	1.33	0.53 - 3.36	0.540	-	-	-	-	-	-
**IPSS-R risk group**												
High/Very High vs. Int/Low/Very Low	3.26	1.44 - 7.34	**0.004**	3.40	1.42 – 8.10	**0.006**	-	-	-	3.82	1.55 - 9.42	**0.004**
***CDKN1C* expression**												
Low *vs.* high expression[2]	1.26	0.60 - 2.63	0.534	1.09	0.50 - 2.40	0.822	-	-	-	-	-	-
Numerical values	0.94	0.87 - 1.03	0.207	0.92	0.84 - 1.02	0.109	-	-	-	0.935	0.82 - 1.07	0.333
***ZBTB38* expression**												
Low *vs.* high expression[2]	1.53	0.73 - 3.22	0.261	2.18	0.95 - 4.98	0.065	-	-	-	2.08	0.76 – 5.68	0.153
Numerical values	0.80	0.57 - 1.11	0.182	0.77	0.53 - 1.11	0.163	-	-	-			

Abbreviations: MDS, myelodysplastic syndromes; IPSS-R, Revised International Prognostic Scoring System.

[1]Hazard ratios >1 indicate that the first factor has the poorer outcome.

[2]MDS patients were categorized as low gene expression (below median) and high gene expression (above median).

Supplementary Table 2:

List of antibodies used for expression analyses

Protein	Supplier	Reference
ACTIN	Thermo Fisher Scientific	PA1-183
CASP3	Cell Signaling Technology	9665S
CCNB1	Cell Signaling	12231P
CDC45L	Proteintech	15678-1-AP
CDKN1A/p21	Cell Signaling Technology	2947P
CDKN1C/p57	Abcam	ab13353
Cleaved CASP3	Cell Signaling Technology	9664P
DNMT1	New England Biolabs	M0231L
GAPDH	Abcam	ab9485
H2AX	Bethyl Laboratories	A300–083A
Histone H3 lysine 9 tri-methylation	Abcam	ab8898
LaminB1	Thermo Fisher Scientific	PA1-41074
LC3I-II	Sigma Aldrich	L7543
MCM3	Abcam	ab4460
P62 / SQSTM1	ENZO Life Science	BML-PW9860
PARP1	Abcam	ab32138
Phospho-ATM	Cell Signaling Technology	4526
Phospho-CHK2	Cell Signaling Technology	2661
Phospho-H2AX	Upstate	05-636
RBBP6	Pr. David Pugh	Homemade (ref. 31)
TP53	Abcam	ab1101
Ubiquitin	Abcam	ab7780
USP9X	Bethyl Laboratories	A301-350A
ZBTB33	Abcam	ab12723
ZBTB38	Dr Pierre-Antoine Defossez	Homemade (ref. 31)
ZBTB4	Dr Pierre-Antoine Defossez	Homemade (#120)

Supplementary Table 3:

List of primers used for gene expression analyses

Gene	Primer Forward	Primer Reverse
CDH1	CCCACCACGTACAAGGGTC	CTGGGGTATTGGGGGCATC
CDKN1A	GCGTTTGGAGTGGTAGAAATCT	CCTGTCACTGTCTTGTACCCT
CDKN1B	AACGTGCGAGTGTCTAACGG	CCCTCTAGGGGTTTGTGATTCT
CDKN1C	ACATCCACGATGGAGCGTC	GGAAGTCGTAATCCCAGCGG
DAPK1	ACGTGGATGATTACTACGACACC	TGCTTTTCTCACGGCATTTCT
DNMT1	CCTGACACCTACCGGCTCTT	AGCAGCTTCCTCCTCCTTATT
DNMT3A	TCCATAAAGCAGGGCAAAGA	AGCGGCTCATGTTGGAGAC
DSCR8	GCCTGGACCCAACTTTGTTA	TCTTGAACTGGGAGGTGGAG
GADD45A	GAGAGCAGAAGACCGAAAGGA	CACAACACCACGTTATCGGG
GAPDH	GGGGTCATTGATGGCAACAATA	ATGGGGAAGGTGAAGGTCG
IDH1	TGTGGTAGAGATGCAAGGAGA	TTGGTGACTTGGTCGTTGGTG
IDH2	CGCCACTATGCCGACAAAAG	ACTGCCAGATAATACGGGTCA
KCNQ1	TGTCCACCATCGAGCAGTATG	CCGTCCCGAAGAACACCAC
LIT1	GATCCTATCCAGGCAGCTTCTTCCACA	CATAAGGTAGGTAAGTTTGTGTCCCTG
MAGEA10	TCCTGCACGGTATGAGTTTCT	GATCACTCCCATTTACCTTGGC
MAPK14	TGCCGAAGATGAACTTTGCGA	TCATAGGTCAGGCTTTTCCACT
MYT1	ACTCCAGGCACCGAAGTTTAC	AGAGGCGTCCTTCACCTCA
RBBP6	CCAGATTGCGACATCATTGCTT	ATGCTCTTCCGCGTCTATTTCT
SLC22A18AS	CTGTGTGCTCCGAGGAGAATG	GTGCATGTTCACGTCCTGTCA
TBP	CCACTCACAGACTCTCACAAC	CTGCGGTACAATCCCAGAACT
TET2	GATAGAACCAACCATGTTGAGGG	TGGAGCTTTGTAGCCAGAGGT
TFRC	ACCATTGTCATATACCCGGTTCA	GGCCTTTGTGTTATTGTCAGCAT
TP53	CCGCAGTCAGATCCTAGCG	AATCATCCATTGCTTGGGACG
UCK1	AGTTGCTGGGACAGAACGAG	CTGCCGTCAGGACCTTGTAG
ZBTB38	ACACTTGCCGAGCACTCATAC	GACGAGGGCGATCTATACAACT

Supplementary Table 4:

MDS and AML patient characteristics.

Patients	Number
**MDS**	**55**
Gender	
Male/Female	27/28
Age (years), median (range):	71 (21-87)
WHO 2008 classification	
RA/RARS/del(5q)/RCMD	3/3/0/30
RAEB-1/RAEB-2	10/9
IPSS-R	
Very low risk/low risk/intermediate	5/18/15
High risk/Very High risk	9/4
Not available	4
Cytogenetic risk[1]	
Very good/good	0/41
Intermediate	10
Poor/very poor	0/0
No growth	4
**AML**	**63**
de novo AML/AML-MRC	47/16
Gender	
Male/Female	38/25
Age (years), median (range):	62 (23-90)
BM blasts (%), median (range)	56 (15-98)
Cytogenetic risk[2]	
Good	7
Intermediate/poor	33/14
No growth	9

Abbreviations: MDS, myelodysplastic syndromes; WHO, World Health Organization; RA, refractory anemia; RARS, refractory anemia with ringed sideroblasts; del(5q), MDS with isolated del(5q); RCMD, refractory cytopenia with multilineage dysplasia; RAEB-1, refractory anemia with excess blast-1; RAEB-2, refractory anemia with excess blast-2; IPSS-R, Revised International Prognostic Scoring System; BM, bone marrow; AML, acute myeloid leukemia; AML-MRC, acute myeloid leukemia with myelodysplasia-related changes.

[1]In MDS cohort, karyotype findings included very good: -Y (n=0), good: normal (n=41), del(5q) (n=0); intermediate: +8 (n=1), -7 (n=1), other (n=8); poor: three abnormalities (n=0); and very poor: >3 abnormalities (n=0).

[2]In AML cohort, low risk karyotype included t(8;21) (n=7) and inv(16) (n=0), intermediate risk included normal (n=26), trisomy 8 (n=2) and other abnormalities (n=5), and high risk included complex karyotype (n=8), del(5q) (n=2) and -7 (n=4).

Supplementary Table 5:

Certificates of cell line authentication.

eurofins | Forensik

DAkkS Deutsche Akkreditierungsstelle D-PL-18779-01-00

CERTIFIED ISO 9001:2008 ACCORDING TO

Institut Cochin U1016
Miotto Benoit
24 rue du Faubourg Saint Jacques
75014 Paris
France

04.05.2017

Certificate

Order
By order of Miotto Benoit (Institut Cochin) we were requested to perform a cell line authentication test. Following samples were examined:

Our sample number	Client sample name
CL170503_015	Hela-S3

Method:
DNA was isolated separately from the samples.
Genetic characteristics were determined by PCR-single-locus-technology. 21 independent PCR-systems Amelogenin, D3S1358, D1S1656, D6S1043, D13S317, Penta E, D16S539, D18S51, D2S1338, CSF1PO, Penta D, TH01, vWA, D21S11, D7S820, D5S818, TPOX, D8S1179, D12S391, D19S433 and FGA were investigated (Promega, PowerPlex 21 PCR Kit).
In parallel, positive and negative controls were carried out yielding correct results.

Results:

DNA-System	DNA-criteria Hela-S3 CL170503_015
AM	X, X
D3S1358	15, 18
D1S1656	12, 15
D6S1043	18, 19
D13S317	13.3, 13.3
Penta E	7, 7
D16S539	9, 10
D18S51	16, 16
D2S1338	17, 17
CSF1PO	9, 10
Penta D	8, 15
TH01	7, 7
vWA	16, 18, 20
D21S11	27, 28
D7S820	8, 12
D5S818	11, 12
TPOX	8, 12
D8S1179	12, 13
D12S391	20, 25
D19S433	13, 14
FGA	18, 21

Summary:

The following cell lines could be detected in the online database of the DSMZ (http://www.dsmz.de/de/service/services-human-and-animal-cell-lines/online-str-analysis.html):

Our sample number	Client sample name	DSMZ name
CL170503_015	Hela-S3	HeLa S3

Dr. Burkhard Rolf
Director Forensic Services

M. Bosch

Dr. Michaela Bosch
Project Manager DNA-Forensics

Eurofins Medigenomix Forensik GmbH carries out all analyses with greatest care and on the basis of state of the art scientific knowledge. All results solely refer to the analysed samples. Our expert's reports must not be duplicated in extracts without consent of Eurofins Medigenomix Forensik GmbH.
Cell_line-certificate_eng_V05_21032017

Vorlage_Verwandtschaftsanalyse_EUROFINS_v02_121127

Eurofins Medigenomix Forensik GmbH
Anzinger Str. 7a
D-85560 Ebersberg

Tel. +49 (0) 8092 8289 400
Fax +49 (0) 8092 8289 201
www.eurofins.de/forensik-de
forensik@eurofins.com

Geschäftsführer
Dr. Peter Persigehl
Dr. Bruno Poddevin

HRB 20 12 60
Amtsgericht München

Ust-Id Nr. DE 285161076
Steuernummer 114/116/00159

Institut Cochin U1016
Miotto Benoit
24 rue du Faubourg Saint Jacques
75014 Paris
France

04.05.2017

Certificate

Order
By order of Miotto Benoit (Institut Cochin) we were requested to perform a cell line authentication test. Following samples were examined:

Our sample number	Client sample name
CL170503_016	U2OS

Method:
DNA was isolated separately from the samples.
Genetic characteristics were determined by PCR-single-locus-technology. 21 independent PCR-systems Amelogenin, D3S1358, D1S1656, D6S1043, D13S317, Penta E, D16S539, D18S51, D2S1338, CSF1PO, Penta D, TH01, vWA, D21S11, D7S820, D5S818, TPOX, D8S1179, D12S391, D19S433 and FGA were investigated (Promega, PowerPlex 21 PCR Kit).
In parallel, positive and negative controls were carried out yielding correct results.

Results:

DNA-System	DNA-criteria U2OS CL170503_016
AM	X, X
D3S1358	16, 16
D1S1656	16, 17.3
D6S1043	11, 11
D13S317	13, 13
Penta E	13, 13
D16S539	11, 12
D18S51	12, 14
D2S1338	20, 24
CSF1PO	12, 13
Penta D	9, 9
TH01	6, 9.3
vWA	14, 18
D21S11	31, 31
D7S820	11, 12
D5S818	8, 11
TPOX	11, 12
D8S1179	12, 14
D12S391	19, 20
D19S433	14, 15
FGA	20, 20

Summary:
The following cell lines could be detected in the online database of the DSMZ (http://www.dsmz.de/de/service/services-human-and-animal-cell-lines/online-str-analysis.html):

Our sample number	Client sample name	DSMZ name
CL170503_016	U2OS	U-2-OS

Dr. Burkhard Rolf
Director Forensic Services

Dr. Michaela Bosch
Project Manager DNA-Forensics

Eurofins Medigenomix Forensik GmbH carries out all analyses with greatest care and on the basis of state of the art scientific knowledge. All results solely refer to the analysed samples. Our expert's reports must not be duplicated in extracts without consent of Eurofins Medigenomix Forensik GmbH.
Cell_line-certificate_eng_V05_21032017

Vorlage_Verwandtschaftsanalyse_EUROFINS_v02_121127

Institut Cochin U1016
Miotto Benoit
24 rue du Faubourg Saint Jacques
75014 Paris
France

04.05.2017

Certificate

Order
By order of Miotto Benoit (Institut Cochin) we were requested to perform a cell line authentication test. Following samples were examined:

Our sample number	Client sample name
CL170503_018	MOLM-14

Method:
DNA was isolated separately from the samples.
Genetic characteristics were determined by PCR-single-locus-technology. 21 independent PCR-systems Amelogenin, D3S1358, D1S1656, D6S1043, D13S317, Penta E, D16S539, D18S51, D2S1338, CSF1PO, Penta D, TH01, vWA, D21S11, D7S820, D5S818, TPOX, D8S1179, D12S391, D19S433 and FGA were investigated (Promega, PowerPlex 21 PCR Kit).
In parallel, positive and negative controls were carried out yielding correct results.

Results:

DNA-System	DNA-criteria MOLM-14 CL170503_018
AM	X, Y
D3S1358	15, 15
D1S1656	11, 16
D6S1043	12, 14
D13S317	10, 11
Penta E	18, 19
D16S539	10, 11
D18S51	13, 15
D2S1338	23, 25
CSF1PO	10, 12
Penta D	9, 12
TH01	7, 7
vWA	16, 17
D21S11	30, 31
D7S820	10, 12
D5S818	10, 11
TPOX	8, 8
D8S1179	13, 14
D12S391	15, 19
D19S433	12, 14
FGA	21, 23

Summary:
The following cell lines could be detected in the online database of the DSMZ (http://www.dsmz.de/de/service/services-human-and-animal-cell-lines/online-str-analysis.html):

Our sample number	Client sample name	DSMZ name
CL170503_018	MOLM-14	MOLM-14

Dr. Burkhard Rolf
Director Forensic Services

Dr. Michaela Bosch
Project Manager DNA-Forensics

Eurofins Medigenomix Forensik GmbH carries out all analyses with greatest care and on the basis of state of the art scientific knowledge. All results solely refer to the analysed samples. Our expert's reports must not be duplicated in extracts without consent of Eurofins Medigenomix Forensik GmbH.
Cell_line-certificate_eng_V05_21032017

Vorlage_Verwandtschaftsanalyse_EUROFINS_v02_121127

Eurofins Medigenomix Forensik GmbH
Anzinger Str. 7a
D-85560 Ebersberg

Tel. +49 (0) 8092 8289 400
Fax +49 (0) 8092 8289 201
www.eurofins.de/forensik-de
forensik@eurofins.com

Geschäftsführer
Dr. Peter Persigehl
Dr. Bruno Poddevin

HRB 20 12 60
Amtsgericht München

Ust-Id Nr. DE 285161076
Steuernummer 114/116/00159

eurofins | Forensik

DAkkS Deutsche Akkreditierungsstelle D-PL-18779-01-00

Institut Cochin U1016
Miotto Benoit

24 rue du Faubourg Saint Jacques
75014 Paris
France

04.05.2017

Certificate

Order
By order of Miotto Benoit (Institut Cochin) we were requested to perform a cell line authentication test. Following samples were examined:

Our sample number	Client sample name
CL170503_019	K562

Method:
DNA was isolated separately from the samples.
Genetic characteristics were determined by PCR-single-locus-technology. 21 independent PCR-systems Amelogenin, D3S1358, D1S1656, D6S1043, D13S317, Penta E, D16S539, D18S51, D2S1338, CSF1PO, Penta D, TH01, vWA, D21S11, D7S820, D5S818, TPOX, D8S1179, D12S391, D19S433 and FGA were investigated (Promega, PowerPlex 21 PCR Kit).
In parallel, positive and negative controls were carried out yielding correct results.

Results:

DNA-System	DNA-criteria K562 CL170503_019
AM	X, X
D3S1358	16, 16
D1S1656	15, 16
D6S1043	11, 15
D13S317	8, 8
Penta E	5, 14
D16S539	11, 12
D18S51	15, 16
D2S1338	17, 17
CSF1PO	9, 10
Penta D	9, 13
TH01	9.3, 9.3
vWA	16, 16
D21S11	29, 30, 31
D7S820	9, 11
D5S818	11, 12, 13
TPOX	8, 9
D8S1179	12, 12
D12S391	23, 23
D19S433	14, 14.2
FGA	21, 24

Summary:

The following cell lines could be detected in the online database of the DSMZ (http://www.dsmz.de/de/service/services-human-and-animal-cell-lines/online-str-analysis.html):

Our sample number	Client sample name	DSMZ name
CL170503_019	K562	K-562

Dr. Burkhard Rolf
Director Forensic Services

Dr. Michaela Bosch
Project Manager DNA-Forensics

Eurofins Medigenomix Forensik GmbH carries out all analyses with greatest care and on the basis of state of the art scientific knowledge. All results solely refer to the analysed samples. Our expert's reports must not be duplicated in extracts without consent of Eurofins Medigenomix Forensik GmbH.
Cell_line-certificate_eng_V05_21032017

Vorlage_Verwandtschaftsanalyse_EUROFINS_v02_121127

Eurofins Medigenomix Forensik GmbH
Anzinger Str. 7a
D-85560 Ebersberg

Tel. +49 (0) 8092 8289 400
Fax +49 (0) 8092 8289 201
www.eurofins.de/forensik-de
forensik@eurofins.com

Geschäftsführer
Dr. Peter Persigehl
Dr. Bruno Poddevin

HRB 20 12 60
Amtsgericht München

Ust-Id Nr. DE 285161076
Steuernummer 114/116/00159

Forensik

Institut Cochin U1016
Miotto Benoit
24 rue du Faubourg Saint Jacques
75014 Paris
France

04.05.2017

Certificate

Order
By order of Miotto Benoit (Institut Cochin) we were requested to perform a cell line authentication test. Following samples were examined:

Our sample number	Client sample name
CL170503_020	HCT-116

Method:
DNA was isolated separately from the samples.
Genetic characteristics were determined by PCR-single-locus-technology. 21 independent PCR-systems Amelogenin, D3S1358, D1S1656, D6S1043, D13S317, Penta E, D16S539, D18S51, D2S1338, CSF1PO, Penta D, TH01, vWA, D21S11, D7S820, D5S818, TPOX, D8S1179, D12S391, D19S433 and FGA were investigated (Promega, PowerPlex 21 PCR Kit).
In parallel, positive and negative controls were carried out yielding correct results.

Results:

DNA-System	DNA-criteria<br>HCT-116<br>CL170503_020
AM	X, X
D3S1358	12, 18, 19
D1S1656	13, 14, 15
D6S1043	12, 13, 14
D13S317	10, 10
Penta E	13, 14
D16S539	10, 11, 13, 14, 15
D18S51	15, 16, 17, 18, 19
D2S1338	15, 16
CSF1PO	7, 10
Penta D	9, 13
TH01	8, 8
vWA	17, 22
D21S11	30, 31
D7S820	12, 12.3
D5S818	10, 11, 12
TPOX	8, 9
D8S1179	11, 12, 13, 14
D12S391	17, 22, 23, 24, 25
D19S433	12, 12
FGA	18, 22, 23, 24

Summary:
The following cell lines could be detected in the online database of the DSMZ (http://www.dsmz.de/de/service/services-human-and-animal-cell-lines/online-str-analysis.html):

Our sample number	Client sample name	DSMZ name
CL170503_020	HCT-116	HCT-116

Dr. Burkhard Rolf
Director Forensic Services

Dr. Michaela Bosch
Project Manager DNA-Forensics

Eurofins Medigenomix Forensik GmbH carries out all analyses with greatest care and on the basis of state of the art scientific knowledge. All results solely refer to the analysed samples. Our expert's reports must not be duplicated in extracts without consent of Eurofins Medigenomix Forensik GmbH.
Cell_line-certificate_eng_V05_21032017

Vorlage_Verwandtschaftsanalyse_EUROFINS_v02_121127

Eurofins Medigenomix Forensik GmbH
Anzinger Str. 7a
D-85560 Ebersberg

Tel. +49 (0) 8092 8289 400
Fax +49 (0) 8092 8289 201
www.eurofins.de/forensik-de
forensik@eurofins.com

Geschäftsführer
Dr. Peter Persigehl
Dr. Bruno Poddevin

HRB 20 12 60
Amtsgericht München

Ust-Id Nr. DE 285161076
Steuernummer 114/116/00159
